# Hypertensive disorders of pregnancy and cardiovascular disease risk: a Mendelian randomisation study

**DOI:** 10.1136/heartjnl-2023-323490

**Published:** 2023-12-26

**Authors:** Lena Tschiderer, Yvonne T van der Schouw, Stephen Burgess, Kitty W M Bloemenkamp, Lisa Seekircher, Peter Willeit, Charlotte Onland-Moret, Sanne A E Peters

**Affiliations:** 1 Institute of Health Economics, Medical University of Innsbruck, Innsbruck, Austria; 2 Julius Center for Health Sciences and Primary Care, University Medical Centre Utrecht, Utrecht, The Netherlands; 3 MRC Biostatistics Unit, University of Cambridge, Cambridge, UK; 4 Department of Public Health and Primary Care, University of Cambridge, Cambridge, UK; 5 Heart and Lung Research Institute, University of Cambridge, Cambridge, UK; 6 Department of Obstetrics, Division Women and Baby, Birth Centre, Wilhelmina Children Hospital, University Medical Centre Utrecht, Utrecht, The Netherlands; 7 The George Institute for Global Health, School of Public Health, Imperial College London, London, UK; 8 The George Institute for Global Health, University of New South Wales, Sydney, New South Wales, Australia

**Keywords:** Pregnancy, Hypertension, Myocardial Infarction, Stroke, Genetics

## Abstract

**Objective:**

Observational studies show that hypertensive disorders of pregnancy (HDPs) are related to unfavourable maternal cardiovascular disease (CVD) risk profiles later in life. We investigated whether genetic liability to pre-eclampsia/eclampsia and gestational hypertension is associated with CVD risk factors and occurrence of CVD events.

**Methods:**

We obtained genetic associations with HDPs from a genome-wide association study and used individual participant data from the UK Biobank to obtain genetic associations with CVD risk factors and CVD events (defined as myocardial infarction or stroke). In our primary analysis, we applied Mendelian randomisation using inverse-variance weighted regression analysis in ever pregnant women. In sensitivity analyses, we studied men and nulligravidae to investigate genetic liability to HDPs and CVD risk without the ability to experience the underlying phenotype.

**Results:**

Our primary analysis included 221 155 ever pregnant women (mean age 56.8 (SD 7.9) years) with available genetic data. ORs for CVD were 1.20 (1.02 to 1.41) and 1.24 (1.12 to 1.38) per unit increase in the log odds of genetic liability to pre-eclampsia/eclampsia and gestational hypertension, respectively. Furthermore, genetic liability to HDPs was associated with higher levels of systolic and diastolic blood pressure and younger age at hypertension diagnosis. Sensitivity analyses revealed no statistically significant differences when comparing the findings with those of nulligravidae and men.

**Conclusions:**

Genetic liability to HDPs is associated with higher CVD risk, lower blood pressure levels and earlier hypertension diagnosis. Our study suggests similar findings in ever pregnant women, nulligravidae and men, implying biological mechanisms relating to HDPs are causally related to CVD risk.

**WHAT IS ALREADY KNOWN ON THIS TOPIC**

- It is well-known that women with hypertensive disorders of pregnancy are at higher risk of cardiovascular disease later in life, but the underlying mechanisms are not entirely clear.

**WHAT THIS STUDY ADDS**

- In this Mendelian randomisation study, the ORs for cardiovascular disease were 1.20 (1.02 to 1.41) per unit increase in the log odds of genetic liability to pre-eclampsia/eclampsia and 1.24 (1.12 to 1.38) per unit increase in the log odds of genetic liability to gestational hypertension in ever pregnant women.

**HOW THIS STUDY MIGHT AFFECT RESEARCH, PRACTICE OR POLICY**

- Our study suggests genetic liability to hypertensive disorders of pregnancy to be an indicator for individuals at higher cardiovascular disease risk later in life. Therefore, women with hypertensive disorders of pregnancy should be monitored closely in order to prevent future cardiovascular disease.

## Introduction

Hypertensive disorders of pregnancy (HDPs) affected one in eight hospital deliveries in 2019 and came along with one in four maternal deaths in 2017–2019 in the USA.^1^ They can express as different phenotypes including gestational hypertension (GH), pre-eclampsia/eclampsia and HELLP syndrome.^2^

A range of risk factors have been proposed for HDPs including higher pre-pregnancy body mass index (BMI), pre-pregnancy diabetes, chronic hypertension, autoimmune disease and maternal age.^3 4^ Furthermore, HDPs are related to multiple immediate, short-term and long-term health concerns, which can affect the mother and the fetus and neonate.^5^ Short-term outcomes are, for instance, stillbirth and preterm delivery,^5^ whereas on the long term, HDPs are associated with maternal cardiovascular risk factors after pregnancy including diabetes, hyperlipidaemia and hypertension.^5^ Observational studies have also related HDPs to higher risk of maternal cardiovascular disease (CVD) events later in life,^5^ which has been confirmed in co-sibling analyses^6^ and by a previous Mendelian randomisation (MR) study that was restricted to sex-combined genetic associations with CVD events.^7^

However, the specific biological mechanisms behind HDPs and CVD risk are not entirely clear. As HDPs and CVD share a considerable amount of risk factors, the theory emerged that both phenotypes are expressions of the same disease pathway at different stages in life,^8^ and that pregnancy enables earlier identification of women at higher CVD risk.^9^ Apart from that, HDPs could cause long-term vascular damage leading to higher CVD risk later in life.^9^

To better understand the role of HDPs in the development of CVD, we conducted an MR study of ever pregnant women from the UK Biobank (UKB) with the aim to estimate the relation of genetic liability to pre-eclampsia/eclampsia and GH with CVD events, blood pressure traits, and lipid-related, liver-related and kidney-related cardiovascular risk factors. In sensitivity analyses, we analyse men and nulligravidae to study the role of genetic liability to HDPs in CVD without experiencing the underlying phenotype. Men and nulligravidae have never been pregnant, but they can serve as negative controls for our analyses as they do have information on genetic variants associated with HDPs. This could help understand whether pregnancy-induced effects themselves or biological mechanisms related to HDPs, that is, spillover effects outside pregnancy, are responsible for higher CVD risk.

## Methods

This analysis adheres to the Strengthening the Reporting of Observational Studies in Epidemiology-MR statement (online supplemental table 1).^10^

10.1136/heartjnl-2023-323490.supp1 Supplementary data

### Study design and data sources

The present study included data from the UKB, of which details have been described previously.^11^ Briefly, the UKB is a large-scale prospective study in the general population of the UK, in which over 500 000 individuals aged 40–69 years were recruited between 2006 and 2010.^11^

For the MR analysis, we used individual-level imputed data on genetic variants. Genotyping was performed using the Affymetrix UK BiLEVE Axiom array and the Affymetrix UKB Axiom array.^12 13^ Genotype imputation was based on the Haplotype Reference Consortium and the UK10K haplotype reference panel.^14^

Of the 502 412 UKB participants, we excluded 43 because they withdrew from the study, 15 207 because they had missing genetic data and 1476 women without data on history of pregnancy. Consequently, 485 686 individuals (221 155 ever pregnant women, 41 506 never pregnant women and 223 025 men) contributed to the present analysis.

Specific definitions of HDPs, CVD events and additional variables, and a power analysis are provided in the online supplemental methods.

### Statistical analysis

We summarised categorical variables with number (percentage) and continuous variables with mean (SD), if normally distributed, and with median (IQR) otherwise. We used two-sided statistical tests and deemed p≤0.05 as statistically significant. Analyses were carried out using R version 4.0.5.

### Instrumental variables

Our instrumental variables included single nucleotide polymorphisms (SNPs) for pre-eclampsia/eclampsia and GH identified by a genome-wide association study (GWAS).^15^ We used results from the discovery analysis, which did not include UKB data, allowing two-sample MR.^15^

The GWAS identified 12 SNPs for pre-eclampsia/eclampsia and seven for GH (online supplemental figure 1).^15^ We harmonised summary-level and UKB data. Furthermore, for both exposures, we excluded one palindromic SNP (rs9855086) with allele frequency between 0.45 and 0.55. To identify related traits, we scanned Phenoscanner^16^ (on 14 February 2023) and extracted traits associated with the included SNPs or with proxies in high linkage disequilibrium (R^2^≥0.8) with p≤5×10^−8^ omitting traits identified from the UKB by the Neale lab (http://www.nealelab.is/uk-biobank/). Details on the SNPs included in the present analysis and related traits are provided in online supplemental table 2.

We measured the strength of our instrumental variables based on the F-statistics of each SNP individually, which ranged from 30 to 51 (online supplemental table 3). In addition, we assessed the association between the polygenic risk scores for HDPs and negative control variables including age and socioeconomic status using linear regression adjusting for the first 16 genetic principal components and found no statistically significant relationships (all p≥0.05).

### Primary analysis

Our primary analysis focused on ever pregnant women. To obtain effect sizes on the association between the genetic variants and CVD, we implemented logistic regression adjusting for age and the first 16 genetic principal components.^17^ We performed MR analysis based on the genetic associations with the HDPs (obtained from the GWAS^15^) and the genetic associations with CVDs (obtained from individual participant data from the UKB). We conducted inverse-variance weighted (IVW) regression using the R-package *MendelianRandomization*.^18^ All ORs in MR analyses are reported per unit increase in the log odds of genetic liability to HDPs.

### Secondary analyses

In secondary analyses, we performed MR analyses (based on IVW regression) using cardiovascular risk factors as outcome variables. These included systolic blood pressure (SBP), diastolic blood pressure (DBP), age at hypertension diagnosis, BMI, total cholesterol, high-density lipoprotein cholesterol, triglycerides, low-density lipoprotein cholesterol (LDL-C), lipoprotein(a), apolipoprotein A1, apolipoprotein B, glycated haemoglobin, creatinine, albumin, alanine aminotransferase (ALAT), aspartate aminotransferase (ASAT), gamma-glutamyltransferase (GGT) and C reactive protein. For total cholesterol and LDL-C, we conducted additional analyses restricted to individuals not taking lipid-lowering therapy. For obtaining genetic associations with risk factors, we used linear regression adjusting for age and the first 16 genetic principal components,^17^ and standardised all risk factors to enhance comparisons. The variables triglycerides, lipoprotein(a), creatinine, ALAT, ASAT, GGT and C reactive protein were log-transformed.

Sensitivity analyses are outlined in the online supplemental methods.

## Results

### Characteristics of the study population

Baseline characteristics of the participants are outlined in table 1. Our primary analysis included 221 155 ever pregnant women. Mean age at baseline was 56.8 (SD 7.9) years. Sensitivity analyses included 41 506 never pregnant women and 223 025 men. CVD was reported in 12 077 ever pregnant women (6363 myocardial infarctions; 6419 strokes), in 1707 never pregnant women (827 myocardial infarctions; 976 strokes) and in 27 681 men (19 745 myocardial infarctions; 10 103 strokes).

**Table 1:** Participant characteristics

Characteristic	Ever pregnant women (n=221 155)		Never pregnant women (n=41 506)		Men (n=223 025)	
	N	Mean±SD, median (IQR), N (%)	N	Mean±SD, median (IQR), N (%)	N	Mean±SD, median (IQR), N (%)
Age, years	221 155	56.8±7.9	41 506	54.1±8.2	223 025	56.7±8.2
SBP, mm Hg	208 912	135.6±19.3	38 861	133.2±18.6	210 822	140.9±17.5
DBP, mm Hg	208 915	80.7±10.0	38 861	80.5±10.1	210 824	84.1±10.0
Hypertension	221 073	99 927 (45.2)	41 496	16 535 (39.8)	222 910	129 449 (58.1)
Age at hypertension diagnosis, years	46 885	50.2±10.9	7477	49.1±9.9	62 993	51.2±9.1
Body mass index, kg/m^2^	220 395	27.1±5.1	41 349	26.8±5.6	222 000	27.8±4.2
Smoking status	220 279		41 409		221 832	
Never		129 436 (58.8)		26 517 (64.0)		108 670 (49.0)
Ex		70 999 (32.2)		11 415 (27.6)		85 425 (38.5)
Current		19 844 (9.0)		3477 (8.4)		27 737 (12.5)
Total cholesterol, mmol/L	210 680	5.9±1.1	39 499	5.8±1.1	212 696	5.5±1.1
HDL cholesterol, mmol/L	191 365	1.6±0.4	35 937	1.6±0.4	196 372	1.3±0.3
Triglycerides, mmol/L	210 562	1.4 (1.0–1.9)	39 480	1.2 (0.9–1.8)	212 467	1.7 (1.2–2.4)
LDL cholesterol, mmol/L	210 340	3.6±0.9	39 434	3.6±0.9	212 240	3.5±0.9
Lipoprotein(a), nmol/L	169 002	22.3 (10.0–62.0)	31 680	21.8 (9.7–61.5)	169 540	19.8 (9.2–61.8)
Apolipoprotein A1, g/L	189 749	1.6±0.3	35 520	1.6±0.3	196 081	1.4±0.2
Apolipoprotein B, g/L	209 954	1.0±0.2	39 365	1.0±0.2	211 222	1.0±0.2
HbA1c, mmol/mol	210 073	35.9±5.9	39 449	35.5±6.3	211 920	36.5±7.6
Creatinine, µmol/L	210 583	63.2 (57.1–70.0)	39 486	62.7 (56.7–69.7)	212 578	80.0 (72.5–88.4)
Albumin, g/L	191 436	45.0±2.6	35 948	44.9±2.6	196 490	45.5±2.6
ALAT, U/L	210 670	17.6 (13.9–23.0)	39 503	17.1 (13.5–22.5)	212 523	23.9 (18.4–32.0)
ASAT, U/L	209 940	23.0 (20.0–26.9)	39 370	22.8 (19.8–26.7)	211 850	26.2 (22.6–31.0)
Gamma-GT, U/L	210 593	21.6 (16.2–32.1)	39 484	20.7 (15.5–30.8)	212 560	33.1 (23.7–50.3)
C reactive protein, mg/L	210 317	1.4 (0.7–3.0)	39 428	1.2 (0.6–2.8)	212 125	1.3 (0.7–2.5)

ALAT, alanine aminotransferase; ASAT, aspartate aminotransferase; DBP, diastolic blood pressure; GT, glutamyltransferase; HbA1c, glycated haemoglobin; HDL, high-density lipoprotein; LDL, low-density lipoprotein; SBP, systolic blood pressure.

### CVD risk

Genetic associations with CVD events are shown in online supplemental table 3.

**Figure 1:**
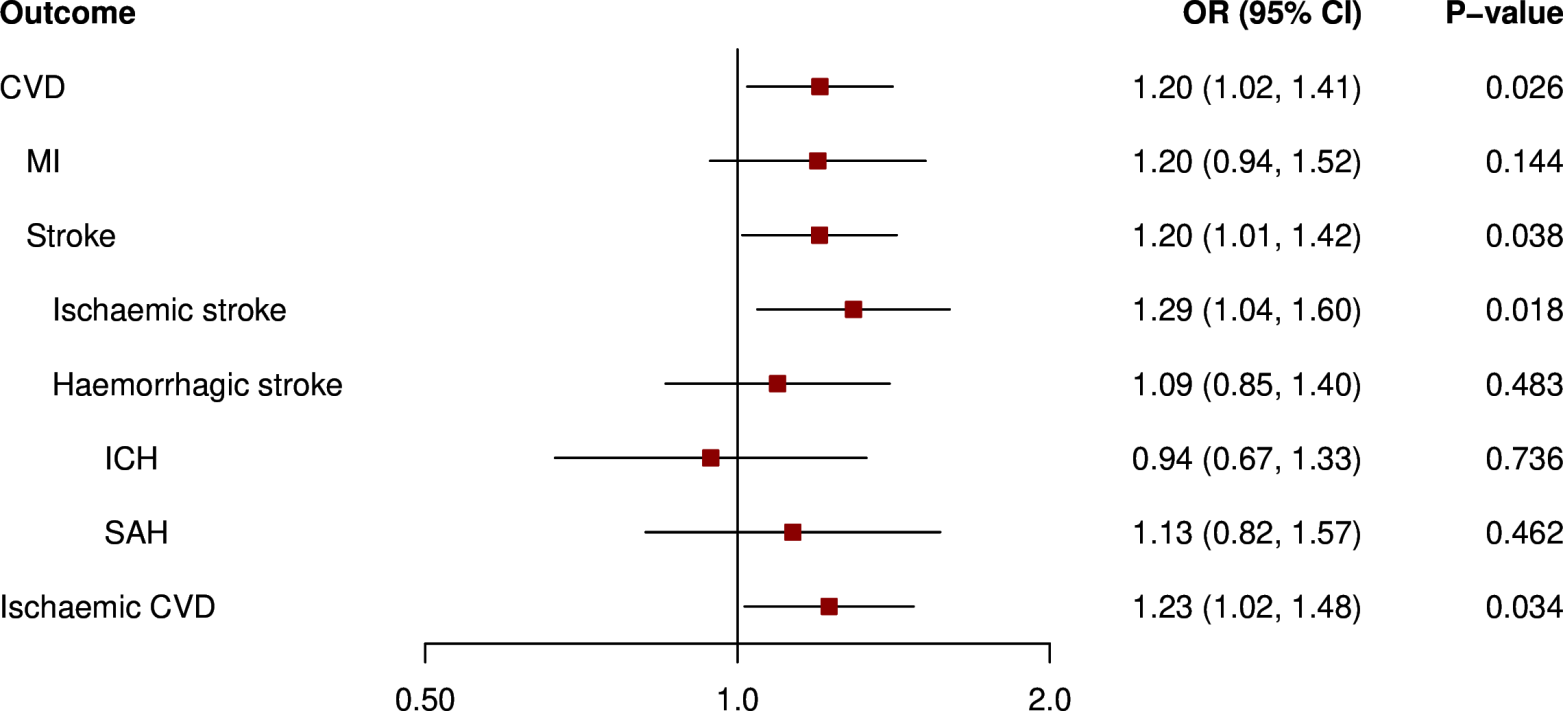
Mendelian randomisation analysis of genetic liability to pre-eclampsia/eclampsia and risk of cardiovascular events in ever pregnant women. Results are from inverse-variance weighted regression. Models were adjusted for age at baseline and the first 16 genetic principal components. CVD, cardiovascular disease; ICH, intracerebral haemorrhage; MI, myocardial infarction; SAH, subarachnoid haemorrhage.

**Figure 2:**
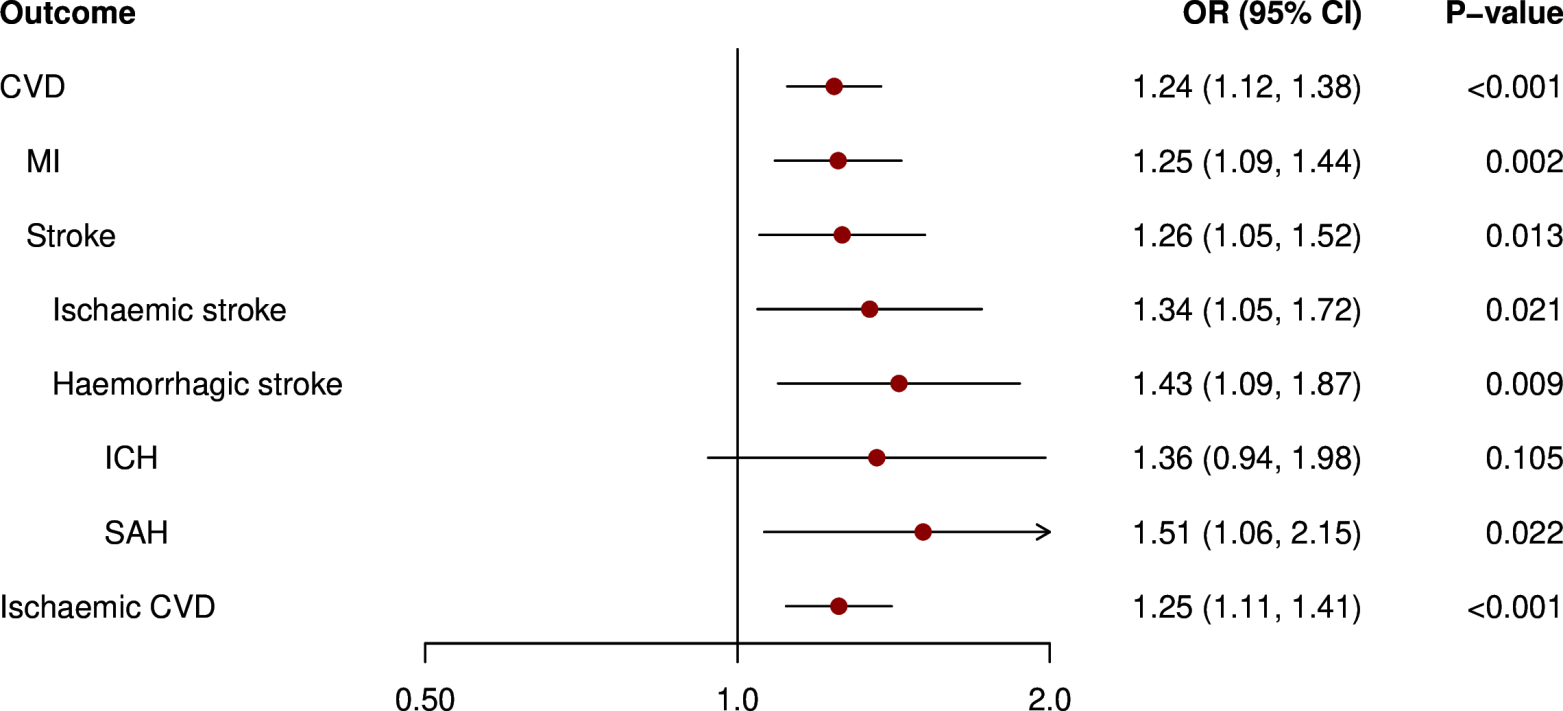
Mendelian randomisation analysis of genetic liability to gestational hypertension and risk of cardiovascular events in ever pregnant women. Results are from inverse-variance weighted regression. Models were adjusted for age at baseline and the first 16 genetic principal components. CVD, cardiovascular disease; ICH, intracerebral haemorrhage; MI, myocardial infarction; SAH, subarachnoid haemorrhage.

As depicted in figure 1, in our primary analysis, genetic liability to pre-eclampsia/eclampsia was related to higher risk of CVD (OR 1.20 (95% CI 1.02 to 1.41)), stroke (1.20 (1.01 to 1.42)), ischaemic stroke (1.29 (1.04 to 1.60)) and ischaemic CVD (1.23 (1.02 to 1.48)). We found no significant association with haemorrhagic stroke, intracerebral haemorrhage (ICH) and subarachnoid haemorrhage (SAH). Moreover, as shown in figure 2, genetic liability to GH was related to a higher risk of CVD (1.24 (1.12 to 1.38)), myocardial infarction (1.25 (1.09 to 1.44)), stroke (1.26 (1.05 to 1.52)), ischaemic stroke (1.34 (1.05 to 1.72)), haemorrhagic stroke (1.43 (1.09 to 1.87)), SAH (1.51 (1.06 to 2.15)) and ischaemic CVD (1.25 (1.11 to 1.41)), but not ICH.

Results were broadly similar when applying different MR methods (online supplemental figures 2 and 3). MR-Egger indicated directional pleiotropy when analysing GH and stroke and ischaemic stroke. In addition, Cochran’s Q-test revealed heterogeneity for the analysis of pre-eclampsia/eclampsia and CVD, myocardial infarction and ischaemic CVD (online supplemental table 4). To investigate this further, we conducted MR-PRESSO,^19^ which detected outlying variants in the analysis of pre-eclampsia/eclampsia and CVD, myocardial infarction and ischaemic CVD. Outlier-corrected results remained directionally similar without statistically significant differences (all p values of distortion tests>0.05) compared with the results of the primary analysis (online supplemental table 5). Findings were somewhat attenuated when applying Cox regression analysis including 216 111 ever pregnant women without history of CVD at baseline (online supplemental figures 4 and 5) and after adjusting our MR analysis for phenotypical SBP (online supplemental figures 6 and 7). Findings were not statistically significantly different from the primary results when analysing nulligravidae and men (online supplemental figures 8–11).

### Blood pressure traits and cardiovascular risk factors

Online supplemental table 3 depicts genetic associations with blood pressure traits and cardiovascular risk factors.

**Figure 3:**
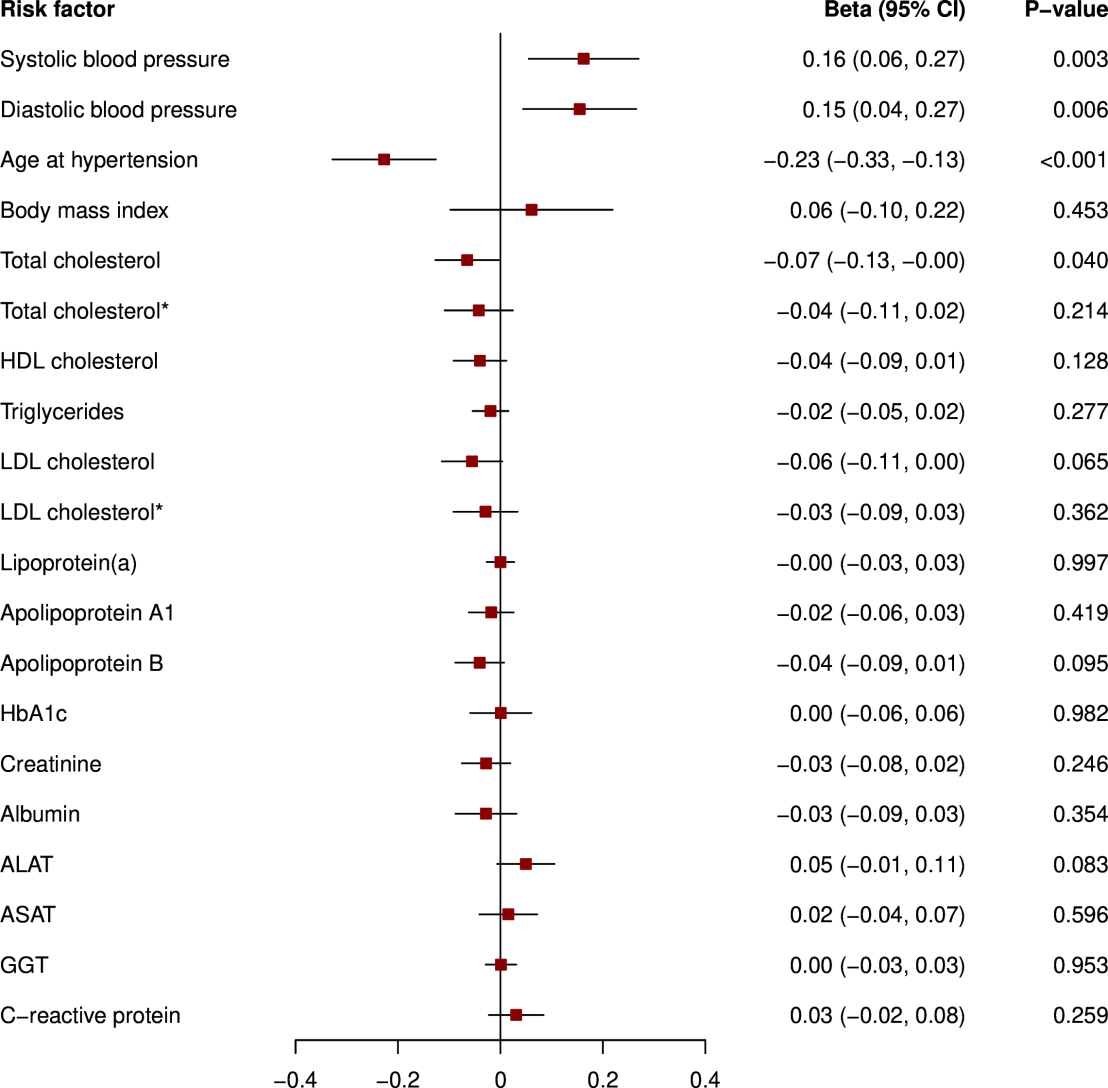
Mendelian randomisation analysis of genetic liability to pre-eclampsia/eclampsia and cardiovascular risk factors in ever pregnant women. *Restricted to individuals not taking lipid-lowering therapy. Results are from inverse-variance weighted regression. Models were adjusted for age at baseline and the first 16 genetic principal components. The variables triglycerides, lipoprotein(a), creatinine, ALAT, ASAT, GGT and C reactive protein were log-transformed. ALAT, alanine aminotransferase; ASAT, aspartate aminotransferase; GGT, gamma-glutamyltransferase; HbA1c, glycated haemoglobin; HDL, high-density lipoprotein; LDL, low-density lipoprotein.

**Figure 4:**
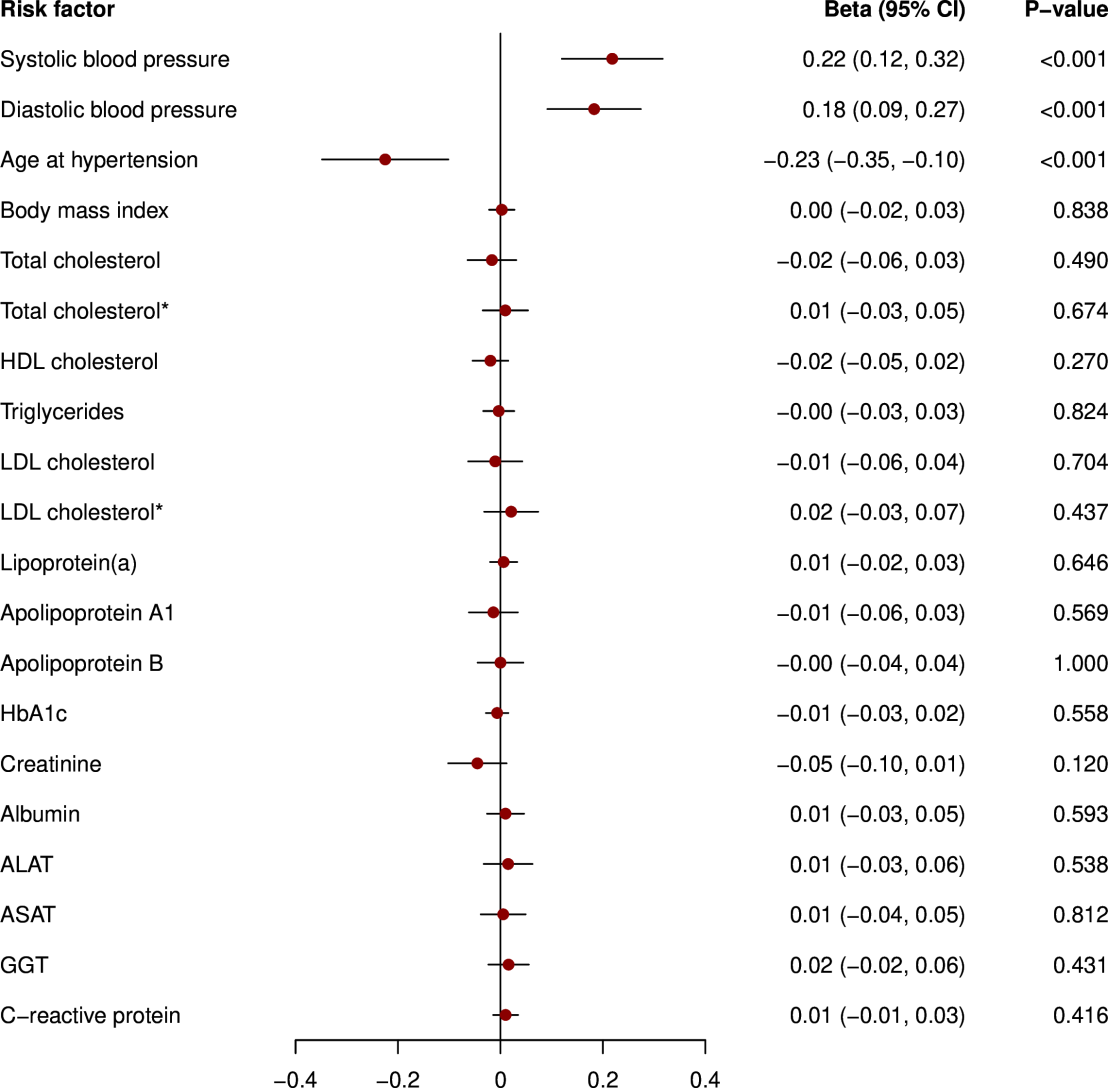
Mendelian randomisation analysis of genetic liability to gestational hypertension and cardiovascular risk factors in ever pregnant women. *Restricted to individuals not taking lipid-lowering therapy. Results are from inverse-variance weighted regression. Models were adjusted for age at baseline and the first 16 genetic principal components. The variables triglycerides, lipoprotein(a), creatinine, ALAT, ASAT, GGT and C reactive protein were log-transformed. ALAT, alanine aminotransferase; ASAT, aspartate aminotransferase; GGT, gamma-glutamyltransferase; HbA1c, glycated haemoglobin; HDL, high-density lipoprotein; LDL, low-density lipoprotein.

Genetic liability to pre-eclampsia/eclampsia was related to higher SBP and DBP levels and earlier hypertension diagnosis in ever pregnant women (figure 3). Furthermore, we found a significant association with lower levels of total cholesterol, which was no longer present after excluding individuals on lipid-lowering medication. Similarly, as depicted in figure 4, genetic liability to GH was associated with higher SBP and DBP levels and younger age at hypertension diagnosis. Again, results were broadly similar when analysing nulligravidae and men (online supplemental figures 12–15).

## Discussion

In this analysis, we found associations between genetic liability to HDPs and higher CVD risk. Furthermore, genetic liability to HDPs was related to higher levels of SBP and DBP and younger age at hypertension diagnosis. We found no statistically significant difference in results for ever pregnant women, nulligravidae and men.

### Findings from previous studies

A large-scale observational meta-analysis showed that moderate and severe pre-eclampsia and GH are associated with a higher risk of maternal CVD.^20^ In a phenome-wide association study, polygenic risk scores of HDPs yielded strong associations with CVD risk factors and CVD events in both sexes.^15^ A recent MR analysis, restricted to women for genetic associations with HDPs, showed genetic liability to HDPs to be related to higher risk of coronary artery disease or ischaemic stroke.^7^ Furthermore, genetic liability to pre-eclampsia and GH were associated with a higher risk of coronary artery disease but not with risk of ischaemic stroke.^7^ This is not in line with the results of the present study. However, the previous MR study relied on a single database for obtaining genetic associations with HDPs and used sex-combined effect sizes for genetic associations with CVD events, which could explain these differences.^7^ In the present MR study, we (1) used effect sizes for the genetic association with the exposures from a large-scale GWAS that meta-analysed results of multiple studies, (2) analysed sex-specific genetic associations (3) included analyses restricted to nulligravidae and men, and (4) studied associations with blood pressure-related, lipid-related, liver-related and kidney-related traits.

### HDPs and blood pressure

Per definition, hypertension is the major component of HDPs. In the present analysis, we showed that genetic liability to HDPs is significantly associated with higher SBP and DBP levels later in life. Genetic correlation between HDPs and blood pressure has previously been demonstrated.^15^ Notably, the genetic correlation between GH and SBP was even higher than between SBP and DBP.^15^ A previous MR analysis showed that higher genetically predicted SBP was related to elevated risk of pre-eclampsia/eclampsia.^21^ Moreover, higher genetically proxied blood pressure levels have been related to an increased risk of CVD in both sexes.^22^ In MR analyses, it is assumed that the genetic instrument can influence the outcome only via the exposure. In case blood pressure lies on the causal pathway between HDPs and CVD, we speak of vertical pleiotropy, which does not bias the findings of our MR analysis. However, we cannot fully exclude horizontal pleiotropy, which would violate the assumptions of MR. When applying MR-Egger, we found no significant intercepts in the relation of HDPs and the majority of CVD outcomes, which indicates absence of directional pleiotropy. We did find a significant MR-Egger intercept in the association between genetic liability to GH and risk of stroke and ischaemic stroke. However, it has previously been discussed that MR-Egger results can be influenced by outlying variants.^23^ When we further studied these associations applying MR-PRESSO, the findings of our primary analysis remained robust. MR also assumes that the genetic instrument is not related to any confounding factors. To check whether phenotypical blood pressure influences our results, we additionally adjusted our MR analysis for phenotypical SBP, which slightly attenuated our results, although they remained directionally robust.

### Ischaemic and haemorrhagic events

In our MR analysis, genetic liability to HDPs was related to ischaemic CVD. Contrarily, we found no statistically significant association between pre-eclampsia/eclampsia and risk of haemorrhagic stroke. These findings are not in line with results from observational studies.^24 25^ This discrepancy could have different reasons. Our analyses may have limited statistical power to analyse haemorrhagic stroke events. Furthermore, observational findings could be affected by confounding. However, women with pre-eclampsia show an excess risk of haemorrhagic stroke during pregnancy.^26^ As our analyses focus on haemorrhagic stroke events during life, including both earlier-life and later-life events, acute haemorrhagic complications due to pregnancy-related hypertension may represent a relatively small proportion of haemorrhagic stroke outcomes and may not be represented adequately in our haemorrhagic stroke endpoint.

### HDPs in men and nulligravidae

In a sensitivity analysis, we also studied men and nulligravidae, who can have the genetic liability to HDPs although the underlying phenotype can never express. We found genetic liability to HDPs to be related to higher CVD risk in men. Robillard *et al* previously suggested the importance of paternity in the development of pre-eclampsia and reported a significantly higher risk of pre-eclampsia in new paternity multiparas compared with same paternity multiparas and primiparas.^27^ To further investigate how genetic liability to HDPs expresses in men, we studied their relationship with a range of cardiovascular risk factors. Genetic liability to HDPs was related to younger age at hypertension diagnosis in men. A recent phenome-wide association study investigated the relation between polygenic risk scores for pre-eclampsia/eclampsia and GH with >1000 phenotypes in men and also reported hypertension to be one of the key associated characteristics.^15^ This is indicative that earlier hypertension is a main driver leading to a higher CVD risk in males. We also analysed the genetic liability to HDPs and CVD in nulligravidae and found no significant differences in effect sizes. However, it should be noted that statistical power for the analysis of nulligravid women was limited. Moreover, the number of pregnancies has been associated with CVD risk in observational studies. In the China Kadoorie Biobank, never pregnant women had a higher risk of CVD than women with one pregnancy.^28^ However, the association was J-shaped, that is, women with a higher number of pregnancies also had a higher risk of CVD compared with women with only one pregnancy.^28^

### Clinical implications

Our MR study demonstrated that genetic liability to HDPs is related to a higher risk of CVD and to higher blood pressure levels and younger age at hypertension diagnosis with similar findings in ever pregnant women, nulligravidae and men. This implies that the causal relationship between genetic liability to HDPs and CVD is not limited to pregnancy-related mechanisms but also to other biological mechanisms. Therefore, HDPs can be an indicator for individuals at higher CVD risk later in life. While pregnancy allows identification of women at increased risk of future CVD, men and nulligravidae with the same genetic liability to HDPs may not have the opportunity to present at an earlier stage in life. Consequently, knowledge about history of HDPs could be implemented in clinical practice. Affected women might have to be monitored closely and other CVD risk factors need to be minimised in order to prevent future CVD. While our analysis suggests that HDPs could be seen as risk signals for later-life CVD, it is still unclear if they also induce processes that promote lasting vascular damage. Therefore, future studies are needed to better understand the driving biological mechanisms behind genetic liability to HDPs which could help us shed more light on the development of CVD.

### Strengths and limitations

Our study has several strengths. We used data from UKB, which provided adequate statistical power to conduct the MR analyses. Furthermore, we built our genetic instruments on a comprehensive large-scale GWAS that meta-analysed data from multiple studies.^15^ Using individual participant data enabled studying sex-specific associations of genetic variants with CVD events. In addition, we conducted several sensitivity analyses including different MR methods and time-to-event analysis. Our study also has limitations. Genetic associations with the exposures were based on individuals of different ancestries (78.0% European), while the UKB majorly includes individuals of European ancestry. Furthermore, we did not have adequate statistical power for analysing nulligravid women, limiting the interpretation of our findings. In addition, we were not able to study more specific phenotypes of HDPs. For instance, epidemiological studies have shown that the association between early-onset pre-eclampsia and CVD risk is specifically strong.^5^ However, to our knowledge, SNPs associated with early-onset pre-eclampsia have not been reported by GWASs so far. Moreover, MR analysis relies on three assumptions. The first assumption is that the instrumental variable is associated with the exposure. Phenotypical data on HDPs were not available in our dataset. Consequently, we were not able to investigate whether the genetic instruments correlated with the phenotype in our study. However, the F-statistics for the included SNPs were ≥30 indicating that we have sufficiently strong instruments. In addition, these SNPs were obtained from an independent GWAS, which replicated the majority of associations in additional cohorts, supporting our assumption of having robust associations with the exposures. The second assumption is that the genetic instrument is not associated with confounders. We studied the association of genetic liability to HDPs with several cardiovascular risk factors. We found significant associations between genetically predicted HDPs and lower levels of lipid parameters. However, these associations were no longer statistically significant after excluding individuals on lipid-lowering medication. The third assumption is that the genetic instrument can influence the outcome only via the exposure. To study this assumption, we conducted MR-Egger regression and MR-PRESSO, which indicated potential directional pleiotropy for single CVD events as discussed in detail above.

## Conclusion

Genetic liability to HDPs including pre-eclampsia/eclampsia and GH is associated with higher CVD risk, lower blood pressure levels and earlier hypertension diagnosis. Our study suggests similar findings in ever pregnant women, nulligravidae and men, implying biological mechanisms relating to these disorders are causally related to CVD risk.

## Data Availability

Data are available in a public, open access repository. Data are available upon reasonable request. Summary-level data from the GWAS have been published previously (doi: 10.1038/s41591-023-02374-9). Data from the UK Biobank can be requested via their website (https://www.ukbiobank.ac.uk/enable-your-research/apply-for-access).
