# Supplementary material for: Hypertensive disorders of pregnancy and cardiovascular disease risk: a Mendelian randomisation study
Source: Heart. 2023 Dec 26;110(10):710–7. doi: 10.1136/heartjnl-2023-323490 (PMC11103302; doi:10.1136/heartjnl-2023-323490)
Supplement: Supplementary data [file heartjnl-2023-323490supp001.pdf]

Supplemental Material

Hypertensive disorders of pregnancy and cardiovascular disease risk: A Mendelian Randomisation study

Tschiderer L, *et al. Heart* 2024; 110:710–717. doi: 10.1136/heartjnl-2023-323490

Supplementary Methods

Definition of pre-eclampsia/eclampsia, gestational hypertension, and CVD events

Pre-eclampsia/eclampsia and gestational hypertension were defined as reported in the genome-wide association study by Honigberg et al.[15] The majority of studies included in their meta-analysis defined pre-eclampsia/eclampsia based on the International Classification of Diseases version 10 (ICD-10) codes O14 and O15 and gestational hypertension based on the ICD-10 code O13.[15] A detailed phenotype definition of pre-eclampsia/eclampsia and gestational hypertension is provided by Honigberg et al.[15]

CVD events were defined according to ICD-10 codes. The combined CVD endpoint was defined as fatal or non-fatal prevalent or incident myocardial infarction (I21, I22, I23, I24.1, I25.2), ischaemic stroke (I63, I64), intracerebral haemorrhage (I61), or subarachnoid haemorrhage (I60). Furthermore, we analysed a combined haemorrhagic stroke endpoint (defined as intracerebral or subarachnoid haemorrhage), a combined stroke endpoint (defined as ischemic or haemorrhagic stroke), and a combined ischaemic CVD endpoint (defined as myocardial infarction or ischaemic stroke). We used September 30th 2021 as end of follow-up for events.

Definition of additional variables

Systolic and diastolic blood pressure were measured using automated reading and were averaged across two measurements taken a few moments apart. Hypertension was defined as self-reported intake of antihypertensive medication or as having systolic blood pressure >140 mmHg or diastolic blood pressure >90 mmHg. Age at hypertension diagnosis was self-reported. BMI was calculated by dividing weight in kg by height in m^2. Smoking status was self-reported and divided into never, ex, and current tobacco smoking. Total cholesterol, high-density lipoprotein cholesterol, low-density lipoprotein cholesterol, triglycerides, apolipoprotein A1, apolipoprotein B, creatinine, albumin, alanine aminotransferase (ALAT) aspartate aminotransferase (ASAT), gamma glutamyltransferase (GGT), and high-sensitivity C-reactive protein were measured on a Beckman Coulter AU5800 platform (assay manufacturer Beckman Coulter [UK], Ltd). Lipoprotein(a) was measured on a Beckman Coulter

Tschiderer L, *et al. Heart* 2024; 110:710–717. doi: 10.1136/heartjnl-2023-323490

AU5800 platform (assay manufacturer Randox Bioscience, UK). Erythrocyte glycated haemoglobin (HbA1c) was measured with Bio-Rad Variant II Turbo analysers. A woman was considered ever pregnant if any livebirth, stillbirth, or pregnancy termination was reported, and nulligravid, otherwise. Since gestational hypertension and pre-eclampsia/eclampsia can only occur from week 20 of gestation, we considered women who reported a miscarriage, but no other pregnancy, as nulligravid. All additional variables were obtained at study baseline, i.e. at time of recruitment into the UKB.

Power analysis

We conducted a power analysis for our primary analysis using an online web tool (https://sb452.shinyapps.io/power/) assuming a sample size of 221,155 and 12,077 CVD cases (as in our Mendelian Randomisation analysis restricted to women and focussing on CVD), a coefficient of determination (R^2) of 0.03 (i.e., the R^2 of the SNPs included in our instrumental variable for gestational hypertension), a causal effect of 1.4 (as reported for gestational hypertension by Garovic et al.[5]), and a significance level of 0.05. We have estimated that such an analysis would have >99.9% power to detect a significant causal effect between genetic liability to gestational hypertension and risk of CVD.

Sensitivity analyses

Sensitivity analyses were conducted similarly to the primary analysis but by applying simple and weighted median regression, MR-Egger, and MR-PRESSO.[19] We also tested differences in estimates before and after excluding potential outliers detected by MR-PRESSO using the MR-PRESSO distortion test.[19] Moreover, we obtained heterogeneity of the primary analysis based on Cochran's Q statistic and the associated P-value that indicates whether all variants estimate the same parameter.

In addition, we performed the same analysis for nulligravid women and men, who do not have the ability the express the phenotype of HDPs. However, they do have the underlying genetic information, i.e., data on genetic associations with HDPs are also available in these populations. This is specifically, important to study whether genetic effects relate only to effects during pregnancy, or if there are spillover effects outside of pregnancy. For these analyses, we additionally obtained ratios of ORs and 95%

Tschiderer L, *et al. Heart* 2024; 110:710–717. doi: 10.1136/heartjnl-2023-323490

CIs by subtracting logORs for women from logORs for men/nulligravid women and summing corresponding variances.

Furthermore, we studied associations between genetic variants and CVD events using a Cox proportional hazards model. We used age as underlying timescale and adjusted the first 16 genetic principal components. For this sensitivity analysis, we excluded all individuals with history of CVD (defined as myocardial infarction or stroke) at baseline. Time-to-event was defined as time to the first CVD event, death, or end of follow-up, whichever occurred first.

In a further sensitivity analysis, we additionally adjusted our MR analyses for phenotypic systolic blood pressure values.

Tschiderer L, *et al. Heart* 2024; 110:710–717. doi: 10.1136/heartjnl-2023-323490

Supplementary Tables

Table S1. STROBE-MR checklist.

Item No.	Section	Checklist item	Page No.
1	**TITLE and ABSTRACT**	Indicate Mendelian randomization (MR) as the study's design in the title and/or the abstract if that is a main purpose of the study	0-1
	**INTRODUCTION**		
2	**Background**	Explain the scientific background and rationale for the reported study. What is the exposure? Is a potential causal relationship between exposure and outcome plausible? Justify why MR is a helpful method to address the study question	3
3	**Objectives**	State specific objectives clearly, including pre-specified causal hypotheses (if any). State that MR is a method that, under specific assumptions, intends to estimate causal effects	3-4
	**METHODS**		
4	**Study design and data sources**	Present key elements of the study design early in the article. Consider including a table listing sources of data for all phases of the study. For each data source contributing to the analysis, describe the following:	
	a)	Setting: Describe the study design and the underlying population, if possible. Describe the setting, locations, and relevant dates, including periods of recruitment, exposure, follow-up, and data collection, when available.	4
	b)	Participants: Give the eligibility criteria, and the sources and methods of selection of participants. Report the sample size, and whether any power or sample size calculations were carried out prior to the main analysis	4
	c)	Describe measurement, quality control and selection of genetic variants	4-6
	d)	For each exposure, outcome, and other relevant variables, describe methods of assessment and diagnostic criteria for diseases	Suppl. Methods
	e)	Provide details of ethics committee approval and participant informed consent, if relevant	4
5	**Assumptions**	Explicitly state the three core IV assumptions for the main analysis (relevance, independence and exclusion restriction) as well assumptions for any additional or sensitivity analysis	11
6	**Statistical methods: main analysis**	Describe statistical methods and statistics used	
	a)	Describe how quantitative variables were handled in the analyses (i.e., scale, units, model)	4-5, Suppl. Methods
	b)	Describe how genetic variants were handled in the analyses and, if applicable, how their weights were selected	4-5
	c)	Describe the MR estimator (e.g. two-stage least squares, Wald ratio) and related statistics. Detail the included covariates and, in case of two-sample MR, whether the same covariate set was used for adjustment in the two samples	5
	d)	Explain how missing data were addressed	4
	e)	If applicable, indicate how multiple testing was addressed	NA
7	**Assessment of assumptions**	Describe any methods or prior knowledge used to assess the assumptions or justify their validity	11
8	**Sensitivity**	Describe any sensitivity analyses or additional analyses performed (e.g.	5-6, Suppl.

	**analyses and additional analyses**	comparison of effect estimates from different approaches, independent replication, bias analytic techniques, validation of instruments, simulations)	Methods
9	**Software and pre-registration**		
	a)	Name statistical software and package(s), including version and settings used	4-5
	b)	State whether the study protocol and details were pre-registered (as well as when and where)	NA
	**RESULTS**		
10	**Descriptive data**		
	a)	Report the numbers of individuals at each stage of included studies and reasons for exclusion. Consider use of a flow diagram	4
	b)	Report summary statistics for phenotypic exposure(s), outcome(s), and other relevant variables (e.g. means, SDs, proportions)	6, Table 1
	c)	If the data sources include meta-analyses of previous studies, provide the assessments of heterogeneity across these studies	doi: 10.1101/2022.11.30.22282929
	d)	For two-sample MR:<br>i. Provide justification of the similarity of the genetic variant-exposure associations between the exposure and outcome samples<br>ii. Provide information on the number of individuals who overlap between the exposure and outcome studies	5
11	**Main results**		
	a)	Report the associations between genetic variant and exposure, and between genetic variant and outcome, preferably on an interpretable scale	doi: 10.1101/2022.11.30.22282929, Table S3
	b)	Report MR estimates of the relationship between exposure and outcome, and the measures of uncertainty from the MR analysis, on an interpretable scale, such as odds ratio or relative risk per SD difference	6-7
	c)	If relevant, consider translating estimates of relative risk into absolute risk for a meaningful time period	NA
	d)	Consider plots to visualize results (e.g. forest plot, scatterplot of associations between genetic variants and outcome versus between genetic variants and exposure)	Fig 1-4
12	**Assessment of assumptions**		
	a)	Report the assessment of the validity of the assumptions	11
	b)	Report any additional statistics (e.g., assessments of heterogeneity across genetic variants, such as I^2, Q statistic or E-value)	6-7
13	**Sensitivity analyses and additional analyses**		
	a)	Report any sensitivity analyses to assess the robustness of the main results to violations of the assumptions	6-7
	b)	Report results from other sensitivity analyses or additional analyses	6-7
	c)	Report any assessment of direction of causal relationship (e.g., bidirectional MR)	6-7

	d)	When relevant, report and compare with estimates from non-MR analyses	8
	e)	Consider additional plots to visualize results (e.g., leave-one-out analyses)	NA
	**DISCUSSION**		
14	**Key results**	Summarize key results with reference to study objectives	7-8
15	**Limitations**	Discuss limitations of the study, taking into account the validity of the IV assumptions, other sources of potential bias, and imprecision. Discuss both direction and magnitude of any potential bias and any efforts to address them	11
16	**Interpretation**		
	a)	Meaning: Give a cautious overall interpretation of results in the context of their limitations and in comparison with other studies	7-8
	b)	Mechanism: Discuss underlying biological mechanisms that could drive a potential causal relationship between the investigated exposure and the outcome, and whether the gene-environment equivalence assumption is reasonable. Use causal language carefully, clarifying that IV estimates may provide causal effects only under certain assumptions	8-10
	c)	Clinical relevance: Discuss whether the results have clinical or public policy relevance, and to what extent they inform effect sizes of possible interventions	10
17	**Generalizability**	Discuss the generalizability of the study results (a) to other populations, (b) across other exposure periods/timings, and (c) across other levels of exposure	11
	**OTHER INFORMATION**		
18	**Funding**	Describe sources of funding and the role of funders in the present study and, if applicable, sources of funding for the databases and original study or studies on which the present study is based	12
19	**Data and data sharing**	Provide the data used to perform all analyses or report where and how the data can be accessed, and reference these sources in the article. Provide the statistical code needed to reproduce the results in the article, or report whether the code is publicly accessible and if so, where	13
20	**Conflicts of Interest**	All authors should declare all potential conflicts of interest	12

Tschiderer L, *et al. Heart* 2024; 110:710–717. doi: 10.1136/heartjnl-2023-323490

Table S2. Details on SNPs used for Mendelian Randomisation analysis.

SNP/trait	PMID
**rs10774624**	
Allergic disease asthma hay fever or eczema	29083406
Basophils	27863252
Basophils, eosinophils	27863252
Basophils, neutrophils	27863252
Beta 2 microglobulin	23417110
BMI	29273807
BP	21909115, 21909110, 19430479
Celiac disease	22057235, 20190752, 21383967
Colorectal cancer	26151821
Colorectal or endometrial cancer	26621817
Coronary artery disease	29212778, 23202125, 19820697, 28714975, 26343387, 28530674
Coronary artery disease age 50	23202125
Cystatin C	20383146
Cystatin C, eGFR	26831199
DBP	21909115, 28739976, 21909110, 27618447, 23202125, 19430479, 21378095, 26390057
Diabetes mellitus type 1	21829393, 19430480, 21980299
Eosinophil percentage of granulocytes	27863252
Eosinophil percentage of white cells	27863252
Eosinophils	27863252, 19198610
Eosinophils, neutrophils	27863252
Fibrinogen	26561523, 28107422
Gene expression of CUX2 in prefrontal cortex	21909115
Gene expression of CUX2 in visual cortex	21909115
Generalized vitiligo	22561518
Glaucoma primary open angle	26752265
Granulocytes	27863252
HDL	24097068
Haematocrit	27863252, 23222517, 19862010
Haemoglobin	27863252, 22139419, 23222517, 28017375, 19862010
Hypertension	21347282, 27618447
Hypothyroidism	22493691
ICAM 1	21533024
IgA deficiency	20694011
Immature fraction of reticulocytes	27863252
Inflammatory bowel disease	26192919
Juvenile idiopathic arthritis	23603761
LDL	24097068, 23063622, 20686565, 23202125
Lymphocytes	27863252, 19198610
Maternal effects on offspring birthweight	29309628
Metabolites	24816252
Monocytes	27863252
Myeloid white cells	27863252
Myocardial infarction	19820697, 26343387

Tschiderer L, *et al. Heart* 2024; 110:710–717. doi: 10.1136/heartjnl-2023-323490

Neutrophils	27863252
Plateletcrit	27863252
Platelets	27863252, 22139419, 19198610, 24026423, 19820697
Primary biliary cirrhosis	22961000
Primary sclerosing cholangitis	27992413, 23603763
RBC	27863252, 23222517, 22139419
Reticulocytes	27863252
Retinal venular caliber	21060863
Rheumatoid arthritis	24390342
Rheumatoid arthritis, celiac disease	21383967
SBP	21909115, 28739976, 21909110, 23202125, 19430479, 21378095
SBP, DBP	19862010
Serum urate	23263486
Tonsillectomy	27182965, 28928442
Total cholesterol	24097068, 23063622, 20686565, 23202125
Vitiligo	27723757
WBC	27863252
**rs13154066**	
Height	25282103, 23754948, 20881960, 23563607
Height (females)	23754948
Height (males)	23754948
Hip circumference	25673412
Waist circumference	25673412
**rs1421085**	
Age at menarche	25231870, 27182965
BMI	29273807, 28892062, 25673413, 20935630, 23001569, 23563607, 28443625, 23754948, 23563609, 28448500, 24861553, 25953783, 19557197, 28270201, 19079261, 21935397, 25044758
BMI (adolescents/young adults)	23669352
BMI (adults)	20421936
BMI (children)	26604143
BMI (females)	25673413, 28443625, 26426971, 28448500, 23754948, 28892062, 23001569
BMI (males)	25673413, 28443625, 26426971, 28892062, 23754948, 28448500, 23001569
BMI age interaction	25953783
BMI smoking interaction	28443625
BMI/obesity	20935630, 19151714, 21935397
BMI/obesity (adults)	20421936, 19151714
BMI/obesity (children)	19151714
Body fat percentage	26833246, 28270201, 20935630
Breast cancer	29059683
Childhood obesity	22484627
C-reactive protein, HDL	27286809
Diabetes mellitus type 2	28566273, 26551672, 24509480, 26818947, 22885922
Dietary macronutrient intake	23636237
Fasting insulin	22885924
HbA1c	28898252

Tschiderer L, *et al. Heart* 2024; 110:710–717. doi: 10.1136/heartjnl-2023-323490

HDL	23063622
Hip circumference	25673412
Hip circumference (females)	25673412
Hip circumference (males)	25673412
Insulin	22885924
Macronutrient intake protein	23636237
Obesity	23563607, 19151714, 20421936
Obesity (early age)	23563609, 20421936, 22484627
Overweight	23563607, 20935630
Waist circumference	25673412, 23754948, 19557161, 19557197
Waist circumference (females)	25673412, 23754948
Waist circumference (males)	25673412, 23754948
Waist hip ratio	25673412, 23754948
Waist hip ratio (females)	25673412
Waist hip ratio (males)	25673412, 23754948
Weight	23754948, 20935630
Weight (females)	23754948
Weight (males)	23754948
**rs149764880**	
Haemoglobin	27863252
Mean corpuscular volume	27863252
Natriuretic peptides	29237677
**rs167479**	
Coronary artery disease	29212778
DBP	27618447
Hypertension	27618447
SBP	27618447
**rs16998073**	
BP	19430483, 21909110, 24954895
Coronary artery disease	29212778, 28714975
DBP	27618447, 19430483, 21378095, 21909115, 21572416
DBP (females)	19430483
Haematocrit	27863252
Haemoglobin	27863252
Hypertension	21347282, 27618447, 19430483
RBC	27863252
SBP	27618447, 21572416, 21909115
**rs2208589**	–
**rs2442752**	
Basophils, eosinophils	27863252
Eosinophils	27863252
Haemoglobin	27863252
IgA deficiency	27723758
Monocytes	27863252
Platelets	27863252
Primary sclerosing cholangitis	27992413
RBC	27863252

Tschiderer L, et al. Heart 2024; 110:710–717. doi: 10.1136/heartjnl-2023-323490

Reticulocytes	27863252
Rheumatoid arthritis	24390342
**rs2508372**	–
**rs259983**	
Coronary artery disease	29212778
Platelets	27863252
**rs260017**	
Coronary artery disease	29212778
Platelets	27863252
**rs5899121**	–
**rs708119**	–
**rs7139122**	–
**rs7318880**	–
**rs9855086**	
Haematocrit	27863252
RBC	27863252

Related traits were identified by Phenoscanner (on February 14th 2023) and extracted all traits associated with the SNPs at P-values≤5×10^{-8} omitting traits identified from the UK Biobank by Neale et al. Abbreviations: BMI, body mass index; BP, blood pressure; eGFR, estimated glomerular filtration rate; DBP, diastolic blood pressure; HbA1c, glycated haemoglobin; HDL, high-density lipoprotein; ICAM, intercellular adhesion molecules; LDL, low-density lipoprotein; RBC, red blood cells; SBP, systolic blood pressure; WBC, white blood cells.

Tschiderer L, *et al. Heart* 2024; 110:710–717. doi: 10.1136/heartjnl-2023-323490

Table S3. Genetic associations with cardiovascular events and cardiovascular risk factors.

SNP	rs10774624	rs13154066	rs1421085	rs149764880	rs167479	rs16998073	rs2208589	rs2442752	rs2508372	rs259983	rs260017	rs5899121	rs708119	rs7139122	rs7318880
**Used for exposure**	PE	GH	PE	PE	PE/GH	PE/GH	GH	PE	PE	PE	GH	PE	PE	GH	PE
**F-statistics**	49	39	48	32	32	42	31	36	37	51	49	30	31	40	47
**Ever pregnant women**															
**CVD**															
Beta	-5.7e-02	1.2e-02	2.9e-03	-4.0e-02	-3.3e-02	2.7e-02	3.3e-02	1.3e-02	8.2e-03	4.4e-02	4.9e-02	-4.2e-03	1.2e-02	-6.3e-03	-2.8e-02
SE	1.4e-02	1.4e-02	1.4e-02	1.8e-02	1.4e-02	1.5e-02	1.6e-02	1.4e-02	1.7e-02	1.9e-02	2.0e-02	2.6e-02	1.5e-02	6.2e-02	1.3e-02
**MI**															
Beta	-8.5e-02	-1.0e-02	-1.0e-02	-2.1e-02	-2.8e-02	3.3e-02	4.0e-02	1.3e-02	-3.2e-02	5.0e-02	5.0e-02	4.9e-03	-2.7e-02	1.0e-01	-4.6e-02
SE	1.9e-02	1.8e-02	1.9e-02	2.5e-02	1.8e-02	2.0e-02	2.2e-02	1.8e-02	2.3e-02	2.6e-02	2.7e-02	3.5e-02	2.0e-02	8.0e-02	1.8e-02
**Stroke**															
Beta	-2.9e-02	3.8e-02	9.0e-03	-3.9e-02	-4.2e-02	3.5e-02	2.7e-02	1.2e-02	3.9e-02	3.2e-02	4.1e-02	-1.6e-02	5.0e-02	-1.3e-01	-2.3e-03
SE	1.9e-02	1.8e-02	1.9e-02	2.5e-02	1.8e-02	2.0e-02	2.2e-02	1.8e-02	2.3e-02	2.6e-02	2.7e-02	3.4e-02	1.9e-02	8.7e-02	1.8e-02
**Ischaemic stroke**															
Beta	-5.1e-02	6.5e-02	2.4e-02	-3.8e-02	-3.4e-02	4.0e-02	2.2e-02	2.3e-03	4.6e-02	7.8e-02	7.5e-02	-5.6e-02	5.1e-02	-1.6e-01	1.9e-02
SE	2.5e-02	2.5e-02	2.5e-02	3.4e-02	2.5e-02	2.7e-02	3.0e-02	2.5e-02	3.1e-02	3.5e-02	3.6e-02	4.6e-02	2.6e-02	1.2e-01	2.5e-02
**Haemorrhagic stroke**															
Beta	6.4e-02	5.3e-02	3.1e-02	-4.3e-02	-4.7e-02	4.2e-02	2.5e-02	-1.8e-02	3.2e-02	1.5e-02	5.0e-02	-2.5e-02	3.0e-02	1.5e-01	7.8e-03
SE	3.6e-02	3.6e-02	3.6e-02	4.8e-02	3.5e-02	3.8e-02	4.3e-02	3.5e-02	4.5e-02	5.1e-02	5.1e-02	6.6e-02	3.8e-02	1.5e-01	3.5e-02
**ICH**															
Beta	5.9e-02	7.2e-02	-6.4e-03	-3.7e-02	-3.4e-02	4.3e-03	5.5e-02	-7.2e-02	9.7e-02	-8.6e-03	3.2e-02	7.6e-02	5.3e-02	6.5e-02	-3.0e-02
SE	5.0e-02	4.9e-02	5.0e-02	6.6e-02	4.9e-02	5.3e-02	6.0e-02	4.9e-02	6.3e-02	7.0e-02	7.1e-02	9.5e-02	5.2e-02	2.1e-01	4.8e-02
**SAH**															
Beta	5.5e-02	1.9e-02	1.9e-02	-4.3e-02	-8.9e-02	4.8e-02	3.6e-02	-3.4e-03	9.3e-03	3.0e-02	5.1e-02	-1.3e-01	4.1e-02	1.9e-01	3.4e-02

Tschiderer L, *et al. Heart* 2024; 110:710–717. doi: 10.1136/heartjnl-2023-323490

SE	4.7e-02	4.7e-02	4.7e-02	6.3e-02	4.6e-02	5.0e-02	5.6e-02	4.6e-02	5.8e-02	6.6e-02	6.8e-02	8.3e-02	4.9e-02	2.0e-01	4.6e-02
**Ischaemic CVD**															
Beta	-7.4e-02	1.2e-02	6.8e-03	-3.8e-02	-2.6e-02	2.8e-02	3.6e-02	5.5e-03	3.0e-03	5.9e-02	5.8e-02	-2.0e-02	-6.6e-04	1.7e-02	-2.9e-02
SE	1.6e-02	1.5e-02	1.6e-02	2.1e-02	1.5e-02	1.7e-02	1.9e-02	1.5e-02	1.9e-02	2.2e-02	2.2e-02	2.9e-02	1.6e-02	6.9e-02	1.5e-02
**SBP**															
Beta	-2.1e-02	3.6e-02	9.4e-03	-4.6e-02	-2.7e-02	4.0e-02	1.6e-02	8.6e-03	-3.5e-03	2.5e-02	2.7e-02	6.4e-03	9.7e-03	1.6e-02	-7.1e-03
SE	3.1e-03	3.0e-03	3.1e-03	4.0e-03	3.0e-03	3.3e-03	3.6e-03	3.0e-03	3.8e-03	4.3e-03	4.4e-03	5.7e-03	3.2e-03	1.4e-02	3.0e-03
**DBP**															
Beta	-3.8e-02	2.7e-02	7.3e-03	-3.5e-02	-2.5e-02	3.4e-02	1.5e-02	-7.4e-04	-1.2e-03	3.0e-02	3.0e-02	4.6e-04	1.1e-02	-1.7e-02	-5.8e-03
SE	3.1e-03	3.1e-03	3.1e-03	4.1e-03	3.0e-03	3.3e-03	3.7e-03	3.1e-03	3.8e-03	4.4e-03	4.5e-03	5.8e-03	3.3e-03	1.4e-02	3.1e-03
**Age at hypertension**															
Beta	2.7e-02	-3.8e-03	-1.9e-02	2.4e-02	3.8e-02	-4.7e-02	-1.8e-02	-2.0e-03	-2.1e-02	-4.4e-02	-4.5e-02	-1.1e-02	-3.6e-03	-3.6e-02	1.4e-03
SE	6.3e-03	6.3e-03	6.3e-03	8.5e-03	6.2e-03	6.7e-03	7.5e-03	6.2e-03	7.8e-03	8.6e-03	8.9e-03	1.2e-02	6.6e-03	2.7e-02	6.2e-03
**BMI**															
Beta	1.2e-02	3.9e-03	7.2e-02	1.4e-02	-1.3e-03	-4.1e-03	1.6e-03	-3.0e-03	3.0e-03	3.4e-04	2.1e-06	8.3e-03	2.1e-03	-7.9e-03	1.2e-03
SE	3.3e-03	3.2e-03	3.3e-03	4.3e-03	3.2e-03	3.5e-03	3.9e-03	3.2e-03	4.0e-03	4.6e-03	4.8e-03	6.1e-03	3.4e-03	1.5e-02	3.2e-03
**Total cholesterol**															
Beta	2.9e-02	2.0e-03	-9.5e-03	-1.2e-03	-3.1e-03	-1.3e-02	-3.5e-03	-5.5e-03	-2.6e-03	-7.3e-03	-7.1e-03	6.2e-03	-1.7e-03	2.4e-02	-5.6e-04
SE	3.0e-03	3.0e-03	3.1e-03	4.0e-03	3.0e-03	3.3e-03	3.6e-03	3.0e-03	3.8e-03	4.3e-03	4.4e-03	5.7e-03	3.2e-03	1.4e-02	3.0e-03
**Total cholesterol***															
Beta	2.8e-02	2.8e-03	-2.7e-03	-8.0e-03	-9.1e-03	-8.6e-03	5.2e-04	-8.4e-03	-3.3e-03	-2.9e-03	-2.6e-03	5.2e-03	-5.3e-04	1.7e-02	-2.5e-03
SE	3.2e-03	3.2e-03	3.2e-03	4.3e-03	3.2e-03	3.5e-03	3.8e-03	3.2e-03	4.0e-03	4.6e-03	4.7e-03	6.0e-03	3.4e-03	1.5e-02	3.2e-03
**HDL-C**															
Beta	2.1e-02	-5.6e-05	-6.6e-03	2.5e-03	2.7e-03	1.7e-03	-2.6e-03	-4.3e-03	4.7e-03	-1.4e-02	-1.5e-02	7.5e-03	-3.1e-03	1.1e-02	2.2e-03
SE	3.2e-03	3.2e-03	3.3e-03	4.3e-03	3.2e-03	3.5e-03	3.8e-03	3.2e-03	4.0e-03	4.6e-03	4.7e-03	6.0e-03	3.4e-03	1.5e-02	3.2e-03
**Triglycerides**															
Beta	-6.4e-03	2.8e-04	2.1e-03	-4.9e-03	3.3e-03	-5.4e-03	5.7e-03	-1.1e-02	-2.6e-03	-5.6e-03	-3.7e-03	-1.6e-03	-2.4e-03	1.7e-02	-6.5e-03
SE	2.9e-03	2.9e-03	2.9e-03	3.8e-03	2.8e-03	3.1e-03	3.4e-03	2.8e-03	3.6e-03	4.1e-03	4.2e-03	5.3e-03	3.0e-03	1.3e-02	2.8e-03
**LDL-C**															
Beta	2.5e-02	3.5e-03	-1.0e-02	-2.5e-03	-5.4e-03	-1.4e-02	-4.9e-03	-7.3e-04	-4.3e-03	-3.2e-03	-2.6e-03	2.8e-03	-7.1e-04	2.0e-02	-1.0e-03

Tschiderer L, *et al. Heart* 2024; 110:710–717. doi: 10.1136/heartjnl-2023-323490

SE	3.1e-03	3.1e-03	3.1e-03	4.1e-03	3.1e-03	3.3e-03	3.7e-03	3.1e-03	3.9e-03	4.4e-03	4.6e-03	5.8e-03	3.3e-03	1.4e-02	3.1e-03
**LDL-C***															
Beta	2.4e-02	5.3e-03	-3.6e-03	-9.3e-03	-1.2e-02	-9.5e-03	-2.7e-04	-2.4e-03	-5.2e-03	2.5e-03	3.0e-03	1.1e-03	4.1e-04	1.4e-02	-2.8e-03
SE	3.3e-03	3.3e-03	3.3e-03	4.4e-03	3.2e-03	3.6e-03	3.9e-03	3.3e-03	4.1e-03	4.7e-03	4.8e-03	6.2e-03	3.5e-03	1.5e-02	3.3e-03
**Lp(a)**															
Beta	2.5e-03	5.6e-03	7.4e-04	-1.0e-04	-2.4e-03	-1.7e-03	-9.2e-04	6.4e-03	6.9e-03	-5.7e-03	-5.5e-03	-1.2e-02	1.4e-03	1.1e-02	-2.6e-04
SE	3.5e-03	3.5e-03	3.5e-03	4.6e-03	3.4e-03	3.7e-03	4.1e-03	3.4e-03	4.3e-03	4.9e-03	5.1e-03	6.4e-03	3.7e-03	1.6e-02	3.4e-03
**Apolipoprotein A1**															
Beta	1.1e-02	2.9e-03	-3.2e-03	-8.5e-03	5.6e-03	4.6e-03	1.0e-04	-2.1e-03	2.7e-03	-1.6e-02	-1.8e-02	8.4e-03	-6.1e-03	1.1e-02	-1.8e-03
SE	3.2e-03	3.2e-03	3.2e-03	4.3e-03	3.2e-03	3.5e-03	3.8e-03	3.2e-03	4.0e-03	4.6e-03	4.7e-03	6.0e-03	3.4e-03	1.5e-02	3.2e-03
**Apolipoprotein B**															
Beta	2.0e-02	3.6e-03	-5.9e-03	-3.8e-03	-5.7e-03	-1.1e-02	-3.7e-03	-7.1e-04	-3.7e-03	-9.2e-04	6.2e-04	-1.2e-03	-1.2e-04	1.9e-02	-2.1e-03
SE	3.1e-03	3.1e-03	3.1e-03	4.1e-03	3.1e-03	3.3e-03	3.7e-03	3.1e-03	3.9e-03	4.4e-03	4.6e-03	5.8e-03	3.3e-03	1.4e-02	3.1e-03
**HbA1c**															
Beta	1.5e-02	-3.7e-04	1.4e-02	-6.1e-03	2.5e-03	-3.3e-03	5.0e-03	1.3e-02	-3.4e-03	1.4e-03	-1.5e-04	6.9e-03	2.6e-03	-1.5e-02	-7.7e-03
SE	2.6e-03	2.6e-03	2.7e-03	3.5e-03	2.6e-03	2.8e-03	3.1e-03	2.6e-03	3.3e-03	3.8e-03	3.9e-03	4.9e-03	2.8e-03	1.2e-02	2.6e-03
**Creatinine**															
Beta	-4.6e-03	-6.4e-03	1.2e-03	8.2e-03	9.4e-03	-1.7e-02	6.4e-04	7.8e-03	1.9e-03	2.5e-03	6.3e-03	-1.6e-04	5.8e-03	-6.4e-03	-2.9e-03
SE	2.5e-03	2.5e-03	2.6e-03	3.4e-03	2.5e-03	2.7e-03	3.0e-03	2.5e-03	3.1e-03	3.6e-03	3.7e-03	4.7e-03	2.7e-03	1.1e-02	2.5e-03
**Albumin**															
Beta	1.6e-02	7.3e-03	-1.6e-02	-1.3e-02	4.5e-03	5.3e-03	-3.5e-03	-3.1e-03	-7.1e-03	-4.3e-03	-1.2e-03	-4.9e-03	-2.6e-03	1.5e-02	9.9e-03
SE	3.3e-03	3.2e-03	3.3e-03	4.3e-03	3.2e-03	3.5e-03	3.9e-03	3.2e-03	4.0e-03	4.6e-03	4.8e-03	6.1e-03	3.4e-03	1.5e-02	3.2e-03
**ALAT**															
Beta	-1.9e-02	1.2e-02	1.4e-02	-1.3e-02	6.2e-03	3.1e-03	-1.2e-04	5.3e-03	-1.1e-02	4.6e-03	5.9e-03	4.6e-03	-3.3e-03	-1.5e-02	-1.0e-03
SE	2.9e-03	2.9e-03	2.9e-03	3.8e-03	2.8e-03	3.1e-03	3.4e-03	2.9e-03	3.6e-03	4.1e-03	4.2e-03	5.4e-03	3.1e-03	1.3e-02	2.8e-03
**ASAT**															
Beta	-2.0e-02	1.0e-02	5.5e-03	-8.4e-03	4.5e-03	1.6e-03	-3.7e-03	-6.9e-03	-1.1e-02	3.5e-03	4.6e-03	-1.0e-02	-2.3e-03	-1.9e-02	-2.9e-03
SE	2.9e-03	2.9e-03	2.9e-03	3.9e-03	2.9e-03	3.1e-03	3.5e-03	2.9e-03	3.6e-03	4.1e-03	4.3e-03	5.4e-03	3.1e-03	1.3e-02	2.9e-03
**GGT**															
Beta	2.2e-03	1.0e-02	7.9e-03	-5.9e-03	4.9e-03	-8.9e-04	3.6e-03	-5.3e-03	-5.0e-03	6.2e-03	4.7e-03	5.1e-03	3.9e-04	-5.1e-03	-4.8e-04

SE	2.9e-03	2.9e-03	2.9e-03	3.8e-03	2.8e-03	3.1e-03	3.4e-03	2.8e-03	3.6e-03	4.1e-03	4.2e-03	5.4e-03	3.1e-03	1.3e-02	2.8e-03
**CRP**															
Beta	5.5e-03	2.2e-03	2.3e-02	-7.0e-03	4.6e-05	4.1e-04	2.7e-03	2.7e-03	-1.5e-03	4.3e-03	5.3e-03	1.5e-02	3.8e-03	-1.3e-02	-2.5e-03
SE	3.2e-03	3.2e-03	3.2e-03	4.2e-03	3.1e-03	3.4e-03	3.8e-03	3.1e-03	3.9e-03	4.5e-03	4.7e-03	5.9e-03	3.4e-03	1.4e-02	3.1e-03
**Never pregnant women**															
**CVD**															
Beta	-1.0e-01	4.6e-02	-2.9e-02	-1.2e-02	-5.7e-02	1.5e-03	-3.8e-02	4.0e-02	3.3e-03	1.2e-02	2.6e-02	8.0e-02	-6.0e-03	-1.4e-01	-5.5e-02
SE	3.6e-02	3.6e-02	3.6e-02	4.8e-02	3.5e-02	3.9e-02	4.3e-02	3.6e-02	4.5e-02	5.2e-02	5.3e-02	6.9e-02	3.8e-02	1.8e-01	3.5e-02
**MI**															
Beta	-1.8e-01	1.1e-02	-8.3e-02	-3.4e-02	-9.1e-02	3.2e-03	-1.2e-02	1.5e-02	2.6e-02	2.4e-02	4.9e-02	1.9e-01	1.7e-02	-1.4e-01	-5.8e-03
SE	5.1e-02	5.1e-02	5.2e-02	6.9e-02	5.0e-02	5.5e-02	6.1e-02	5.1e-02	6.4e-02	7.3e-02	7.5e-02	1.0e-01	5.4e-02	2.6e-01	5.0e-02
**Stroke**															
Beta	-6.0e-02	8.7e-02	2.0e-02	1.2e-02	-1.3e-02	-3.7e-03	-8.2e-02	2.5e-02	-2.7e-02	4.7e-03	2.0e-02	-5.2e-02	-3.5e-02	-9.7e-02	-9.4e-02
SE	4.7e-02	4.7e-02	4.7e-02	6.2e-02	4.6e-02	5.1e-02	5.5e-02	4.7e-02	5.8e-02	6.7e-02	6.9e-02	8.6e-02	5.0e-02	2.3e-01	4.6e-02
**Ischaemic stroke**															
Beta	-1.6e-01	5.5e-02	1.6e-02	-6.9e-02	5.4e-02	-3.9e-02	-1.2e-01	8.5e-02	-1.1e-02	4.3e-02	5.1e-02	1.7e-02	-9.0e-02	-2.6e-01	-6.4e-02
SE	6.3e-02	6.3e-02	6.3e-02	8.5e-02	6.1e-02	6.8e-02	7.3e-02	6.2e-02	7.7e-02	8.8e-02	9.1e-02	1.2e-01	6.8e-02	3.3e-01	6.1e-02
**Haemorrhagic stroke**															
Beta	1.5e-01	1.2e-01	-1.1e-02	1.7e-02	-4.1e-02	5.0e-02	-1.7e-01	-9.8e-02	-9.5e-03	7.5e-04	3.2e-03	-3.2e-02	-1.0e-01	3.3e-02	-4.7e-02
SE	9.0e-02	9.1e-02	9.0e-02	1.2e-01	8.8e-02	9.6e-02	1.0e-01	9.0e-02	1.1e-01	1.3e-01	1.3e-01	1.6e-01	9.7e-02	4.2e-01	8.8e-02
**ICH**															
Beta	1.9e-01	1.2e-01	-1.2e-02	-3.5e-02	-9.7e-02	4.0e-02	-2.2e-01	-3.8e-03	3.0e-01	1.5e-01	1.3e-01	-7.8e-02	-3.2e-01	5.8e-02	-1.2e-01
SE	1.3e-01	1.3e-01	1.3e-01	1.7e-01	1.3e-01	1.4e-01	1.5e-01	1.3e-01	1.7e-01	1.7e-01	1.8e-01	2.3e-01	1.5e-01	5.9e-01	1.3e-01
**SAH**															
Beta	9.5e-02	6.2e-02	3.8e-02	6.1e-02	1.9e-02	7.7e-02	-1.3e-01	-1.4e-01	-1.9e-01	-2.2e-01	-1.9e-01	4.6e-02	5.4e-02	-1.1e-01	-3.1e-02
SE	1.2e-01	1.2e-01	1.2e-01	1.5e-01	1.2e-01	1.2e-01	1.4e-01	1.2e-01	1.4e-01	1.8e-01	1.9e-01	2.2e-01	1.2e-01	6.0e-01	1.2e-01
**Ischaemic CVD**															
Beta	-1.6e-01	2.7e-02	-4.6e-02	-4.3e-02	-4.3e-02	-3.5e-03	-3.2e-02	5.2e-02	8.5e-03	3.0e-02	4.7e-02	1.2e-01	-2.1e-02	-2.4e-01	-3.0e-02
SE	4.1e-02	4.1e-02	4.1e-02	5.5e-02	4.0e-02	4.4e-02	4.9e-02	4.1e-02	5.1e-02	5.8e-02	6.0e-02	8.0e-02	4.4e-02	2.2e-01	4.0e-02
**SBP**															

Tschiderer L, *et al. Heart* 2024; 110:710–717. doi: 10.1136/heartjnl-2023-323490

Beta	-2.2e-02	4.2e-02	1.1e-02	-3.3e-02	-2.7e-02	3.5e-02	2.4e-02	2.6e-03	-2.5e-03	1.8e-02	2.2e-02	1.4e-02	7.3e-03	-4.0e-02	-7.8e-03
SE	6.8e-03	6.8e-03	6.8e-03	9.1e-03	6.7e-03	7.4e-03	8.1e-03	6.7e-03	8.4e-03	9.8e-03	1.0e-02	1.3e-02	7.2e-03	3.2e-02	6.7e-03
**DBP**															
Beta	-4.8e-02	3.9e-02	3.0e-03	-2.4e-02	-3.2e-02	3.8e-02	2.4e-02	-4.0e-03	7.9e-03	2.5e-02	2.8e-02	1.0e-02	1.0e-02	-1.7e-02	-1.2e-02
SE	7.3e-03	7.3e-03	7.3e-03	9.7e-03	7.2e-03	7.9e-03	8.7e-03	7.2e-03	9.0e-03	1.0e-02	1.1e-02	1.4e-02	7.7e-03	3.4e-02	7.2e-03
**Age at hypertension**															
Beta	3.5e-02	-1.4e-02	-3.4e-02	8.9e-03	1.1e-02	-2.3e-02	6.7e-03	-1.0e-02	1.4e-02	-4.2e-02	-5.6e-02	-3.1e-02	-3.7e-02	4.0e-02	-1.4e-02
SE	1.3e-02	1.3e-02	1.3e-02	1.8e-02	1.3e-02	1.4e-02	1.6e-02	1.3e-02	1.6e-02	1.8e-02	1.9e-02	2.4e-02	1.4e-02	6.0e-02	1.3e-02
**BMI**															
Beta	1.6e-02	9.4e-03	1.0e-01	1.5e-02	1.0e-02	-1.7e-02	7.6e-03	-5.9e-03	2.2e-02	-2.7e-03	3.1e-03	3.5e-02	9.1e-03	8.4e-02	3.2e-03
SE	8.3e-03	8.2e-03	8.3e-03	1.1e-02	8.1e-03	8.9e-03	9.8e-03	8.2e-03	1.0e-02	1.2e-02	1.2e-02	1.5e-02	8.8e-03	3.9e-02	8.1e-03
**Total cholesterol**															
Beta	1.5e-02	-4.9e-03	-2.5e-03	5.0e-03	1.0e-02	-1.1e-02	2.3e-03	-1.6e-02	1.6e-02	-1.6e-03	-2.2e-03	-3.9e-03	2.2e-03	1.0e-02	4.2e-03
SE	6.9e-03	6.8e-03	6.9e-03	9.1e-03	6.7e-03	7.4e-03	8.1e-03	6.8e-03	8.4e-03	9.8e-03	1.0e-02	1.3e-02	7.3e-03	3.2e-02	6.7e-03
**Total cholesterol***															
Beta	1.2e-02	-6.0e-03	3.9e-03	5.1e-04	5.2e-03	-5.6e-03	3.5e-03	-1.1e-02	1.5e-02	3.9e-03	5.5e-03	-1.8e-03	-2.2e-04	4.2e-03	3.3e-03
SE	7.3e-03	7.3e-03	7.3e-03	9.7e-03	7.2e-03	7.9e-03	8.7e-03	7.2e-03	9.0e-03	1.0e-02	1.1e-02	1.4e-02	7.7e-03	3.4e-02	7.2e-03
**HDL-C**															
Beta	2.6e-02	-4.6e-03	-7.1e-03	3.7e-03	3.1e-03	3.4e-03	8.5e-04	-1.4e-02	-4.4e-03	-1.0e-02	-1.8e-02	-2.1e-02	-6.4e-03	-1.1e-02	5.5e-03
SE	7.8e-03	7.8e-03	7.8e-03	1.0e-02	7.7e-03	8.4e-03	9.2e-03	7.7e-03	9.6e-03	1.1e-02	1.1e-02	1.4e-02	8.3e-03	3.7e-02	7.6e-03
**Triglycerides**															
Beta	-1.5e-02	4.0e-03	3.9e-03	2.9e-04	2.0e-02	-7.8e-03	1.5e-03	-1.3e-02	1.0e-02	2.2e-03	3.8e-03	-6.2e-04	6.0e-03	5.5e-03	2.5e-03
SE	6.7e-03	6.7e-03	6.7e-03	8.9e-03	6.6e-03	7.3e-03	8.0e-03	6.6e-03	8.3e-03	9.6e-03	9.9e-03	1.2e-02	7.1e-03	3.2e-02	6.6e-03
**LDL-C**															
Beta	8.2e-03	-4.9e-03	-3.6e-03	2.4e-03	7.5e-03	-1.1e-02	2.3e-03	-8.9e-03	1.6e-02	4.0e-03	5.4e-03	5.2e-03	3.6e-03	2.0e-02	1.5e-03
SE	7.1e-03	7.0e-03	7.0e-03	9.3e-03	6.9e-03	7.6e-03	8.3e-03	6.9e-03	8.6e-03	1.0e-02	1.0e-02	1.3e-02	7.5e-03	3.3e-02	6.9e-03
**LDL-C***															
Beta	4.1e-03	-6.3e-03	3.3e-03	-2.9e-03	1.5e-03	-6.4e-03	2.9e-03	-2.7e-03	1.6e-02	1.0e-02	1.4e-02	7.4e-03	2.7e-03	1.6e-02	7.3e-04
SE	7.5e-03	7.5e-03	7.5e-03	9.9e-03	7.3e-03	8.1e-03	8.9e-03	7.4e-03	9.2e-03	1.1e-02	1.1e-02	1.4e-02	7.9e-03	3.5e-02	7.3e-03
**Lp(a)**															

Tschiderer L, *et al. Heart* 2024; 110:710–717. doi: 10.1136/heartjnl-2023-323490

Beta	-4.1e-03	-4.6e-04	1.4e-02	-1.7e-03	-5.5e-03	3.8e-03	-1.0e-04	1.8e-03	-7.7e-04	5.2e-03	-3.9e-03	1.5e-02	1.2e-02	-3.4e-02	7.0e-03
SE	8.1e-03	8.0e-03	8.1e-03	1.1e-02	7.9e-03	8.7e-03	9.6e-03	8.0e-03	9.9e-03	1.2e-02	1.2e-02	1.5e-02	8.6e-03	3.8e-02	7.9e-03
**Apolipoprotein A1**															
Beta	1.7e-02	-1.8e-03	-7.7e-03	-7.3e-03	1.3e-02	5.6e-03	6.4e-03	-9.3e-03	-6.9e-03	-3.2e-03	-6.9e-03	-3.2e-02	-6.8e-03	-1.7e-02	-2.3e-03
SE	7.7e-03	7.7e-03	7.7e-03	1.0e-02	7.6e-03	8.3e-03	9.1e-03	7.6e-03	9.5e-03	1.1e-02	1.1e-02	1.4e-02	8.2e-03	3.6e-02	7.6e-03
**Apolipoprotein B**															
Beta	4.8e-03	-2.9e-03	-3.0e-03	-1.6e-03	7.7e-03	-6.8e-03	-3.2e-03	-6.5e-03	1.3e-02	8.3e-03	1.1e-02	8.4e-03	8.3e-03	2.0e-02	1.9e-03
SE	7.1e-03	7.0e-03	7.1e-03	9.4e-03	6.9e-03	7.6e-03	8.4e-03	7.0e-03	8.7e-03	1.0e-02	1.0e-02	1.3e-02	7.5e-03	3.3e-02	6.9e-03
**HbA1c**															
Beta	6.1e-03	1.1e-03	2.3e-02	-1.4e-02	-5.7e-03	7.3e-03	1.9e-02	2.3e-02	-1.1e-02	5.8e-03	2.7e-03	8.1e-03	-2.1e-03	-1.3e-02	5.4e-03
SE	6.6e-03	6.6e-03	6.6e-03	8.8e-03	6.5e-03	7.1e-03	7.8e-03	6.5e-03	8.1e-03	9.4e-03	9.7e-03	1.2e-02	7.0e-03	3.1e-02	6.5e-03
**Creatinine**															
Beta	-6.6e-03	-4.4e-03	5.1e-04	9.0e-03	1.6e-02	-2.0e-02	2.0e-03	9.1e-03	-8.1e-03	9.5e-03	1.5e-02	-2.6e-04	-1.3e-04	-4.4e-02	-4.0e-03
SE	6.1e-03	6.1e-03	6.1e-03	8.1e-03	6.0e-03	6.6e-03	7.2e-03	6.0e-03	7.5e-03	8.7e-03	9.0e-03	1.1e-02	6.5e-03	2.9e-02	6.0e-03
**Albumin**															
Beta	2.0e-02	8.3e-03	-1.5e-02	-1.0e-02	4.4e-03	9.7e-03	-3.1e-03	3.8e-03	7.1e-03	-7.5e-03	-2.0e-03	-1.1e-02	1.4e-03	-2.9e-02	5.9e-03
SE	7.7e-03	7.6e-03	7.7e-03	1.0e-02	7.5e-03	8.3e-03	9.1e-03	7.6e-03	9.4e-03	1.1e-02	1.1e-02	1.4e-02	8.2e-03	3.6e-02	7.5e-03
**ALAT**															
Beta	-2.1e-02	-2.4e-03	2.2e-02	-1.3e-02	6.7e-03	2.5e-03	-1.3e-02	-5.4e-03	-1.2e-03	2.7e-03	7.1e-03	1.9e-02	3.5e-03	-2.7e-02	3.3e-03
SE	6.8e-03	6.7e-03	6.8e-03	9.0e-03	6.6e-03	7.3e-03	8.0e-03	6.7e-03	8.3e-03	9.7e-03	1.0e-02	1.3e-02	7.2e-03	3.2e-02	6.6e-03
**ASAT**															
Beta	-1.6e-02	4.2e-04	1.1e-02	-7.9e-03	-7.9e-04	-1.4e-03	-8.1e-03	-1.2e-02	-5.8e-03	5.7e-03	8.4e-03	1.1e-02	3.4e-03	-3.3e-02	-4.5e-03
SE	6.8e-03	6.8e-03	6.8e-03	9.0e-03	6.7e-03	7.4e-03	8.1e-03	6.7e-03	8.4e-03	9.7e-03	1.0e-02	1.3e-02	7.2e-03	3.2e-02	6.7e-03
**GGT**															
Beta	1.7e-05	8.6e-03	1.4e-02	-1.3e-02	1.6e-02	-7.4e-04	1.3e-02	-1.5e-02	-3.4e-03	-3.3e-04	4.4e-04	3.2e-03	1.3e-02	2.2e-02	9.9e-04
SE	6.8e-03	6.7e-03	6.8e-03	9.0e-03	6.6e-03	7.3e-03	8.0e-03	6.7e-03	8.3e-03	9.7e-03	1.0e-02	1.3e-02	7.2e-03	3.2e-02	6.6e-03
**CRP**															
Beta	2.2e-03	4.1e-03	3.0e-02	3.4e-03	9.6e-03	-3.2e-03	1.3e-02	1.1e-02	-6.7e-03	-1.6e-03	3.3e-03	-6.5e-03	5.0e-03	1.5e-03	3.4e-03
SE	7.7e-03	7.7e-03	7.7e-03	1.0e-02	7.6e-03	8.3e-03	9.2e-03	7.6e-03	9.5e-03	1.1e-02	1.1e-02	1.4e-02	8.2e-03	3.6e-02	7.6e-03
**Men**															

Tschiderer L, *et al. Heart* 2024; 110:710–717. doi: 10.1136/heartjnl-2023-323490

**CVD**															
Beta	-7.9e-02	3.1e-02	2.8e-03	-3.0e-02	-2.7e-02	4.5e-02	1.4e-02	1.7e-02	-2.3e-02	4.7e-02	5.0e-02	2.2e-02	5.8e-04	3.1e-02	1.8e-03
SE	9.5e-03	9.5e-03	9.5e-03	1.3e-02	9.3e-03	1.0e-02	1.1e-02	9.4e-03	1.2e-02	1.3e-02	1.4e-02	1.8e-02	1.0e-02	4.3e-02	9.3e-03
**MI**															
Beta	-7.9e-02	2.2e-02	9.2e-03	-2.9e-02	-3.5e-02	5.1e-02	2.5e-02	1.2e-02	-1.8e-02	4.9e-02	5.1e-02	3.4e-02	4.8e-03	3.7e-02	6.9e-03
SE	1.1e-02	1.1e-02	1.1e-02	1.5e-02	1.1e-02	1.2e-02	1.3e-02	1.1e-02	1.4e-02	1.5e-02	1.6e-02	2.1e-02	1.2e-02	5.0e-02	1.1e-02
**Stroke**															
Beta	-6.8e-02	2.7e-02	9.3e-03	-3.1e-02	-1.9e-02	3.9e-02	6.7e-04	1.8e-02	-1.9e-02	3.6e-02	4.3e-02	-2.4e-03	-1.9e-02	2.5e-02	-8.5e-03
SE	1.5e-02	1.5e-02	1.5e-02	2.0e-02	1.5e-02	1.6e-02	1.8e-02	1.5e-02	1.8e-02	2.1e-02	2.2e-02	2.8e-02	1.6e-02	6.6e-02	1.5e-02
**Ischaemic stroke**															
Beta	-7.7e-02	2.3e-02	1.6e-02	-3.2e-02	-2.1e-02	6.7e-02	1.7e-02	3.6e-02	-2.6e-02	5.0e-02	5.5e-02	-6.2e-03	-2.2e-02	1.3e-02	-2.1e-02
SE	1.9e-02	1.9e-02	1.9e-02	2.5e-02	1.8e-02	2.0e-02	2.2e-02	1.8e-02	2.3e-02	2.6e-02	2.7e-02	3.5e-02	2.0e-02	8.2e-02	1.8e-02
**Haemorrhagic stroke**															
Beta	1.4e-02	9.4e-02	-4.2e-02	-2.2e-02	-2.1e-02	-1.4e-03	-9.4e-02	3.0e-02	-2.4e-02	4.5e-02	6.7e-02	-3.1e-03	9.5e-02	4.2e-02	-3.3e-02
SE	3.4e-02	3.4e-02	3.4e-02	4.6e-02	3.4e-02	3.7e-02	4.0e-02	3.4e-02	4.2e-02	4.8e-02	4.9e-02	6.4e-02	3.6e-02	1.5e-01	3.4e-02
**ICH**															
Beta	2.3e-02	6.3e-02	-2.1e-02	-2.1e-02	-2.4e-02	-9.3e-04	-1.3e-01	4.3e-02	-4.7e-02	5.5e-02	9.6e-02	1.3e-02	1.2e-01	-1.7e-01	-2.8e-02
SE	4.2e-02	4.2e-02	4.2e-02	5.6e-02	4.1e-02	4.5e-02	4.8e-02	4.1e-02	5.1e-02	5.8e-02	5.9e-02	7.8e-02	4.3e-02	2.0e-01	4.1e-02
**SAH**															
Beta	-1.2e-02	1.5e-01	-1.1e-01	-4.1e-02	-5.8e-03	-5.5e-02	-2.9e-02	3.1e-02	6.7e-03	2.1e-02	1.5e-02	-1.9e-02	5.9e-02	2.7e-01	-2.9e-02
SE	5.5e-02	5.5e-02	5.5e-02	7.4e-02	5.4e-02	6.0e-02	6.5e-02	5.4e-02	6.8e-02	7.7e-02	8.0e-02	1.0e-01	5.7e-02	2.1e-01	5.4e-02
**Ischaemic CVD**															
Beta	-8.3e-02	2.8e-02	7.7e-03	-3.0e-02	-3.0e-02	5.4e-02	2.4e-02	2.0e-02	-2.6e-02	5.1e-02	5.4e-02	2.6e-02	6.7e-04	3.5e-02	1.2e-03
SE	1.0e-02	9.9e-03	9.9e-03	1.3e-02	9.8e-03	1.1e-02	1.2e-02	9.8e-03	1.2e-02	1.4e-02	1.4e-02	1.9e-02	1.1e-02	4.5e-02	9.8e-03
**SBP**															
Beta	-1.7e-02	2.9e-02	6.5e-03	-3.6e-02	-2.3e-02	3.2e-02	1.0e-02	4.4e-03	-3.9e-04	1.6e-02	1.8e-02	1.0e-02	5.6e-04	1.8e-02	-1.8e-03
SE	2.9e-03	2.8e-03	2.9e-03	3.8e-03	2.8e-03	3.1e-03	3.4e-03	2.8e-03	3.5e-03	4.1e-03	4.2e-03	5.3e-03	3.0e-03	1.3e-02	2.8e-03
**DBP**															
Beta	-3.4e-02	2.5e-02	5.8e-03	-3.7e-02	-2.6e-02	3.5e-02	1.6e-02	-4.5e-03	2.1e-03	2.3e-02	2.6e-02	7.4e-03	2.0e-03	-1.9e-02	-3.7e-03
SE	3.1e-03	3.1e-03	3.1e-03	4.1e-03	3.1e-03	3.4e-03	3.7e-03	3.1e-03	3.9e-03	4.4e-03	4.6e-03	5.8e-03	3.3e-03	1.4e-02	3.1e-03

**Age at hypertension**															
Beta	1.9e-02	-1.3e-02	-8.5e-03	2.4e-02	1.8e-02	-3.1e-02	-2.0e-02	3.8e-03	-7.0e-03	-2.6e-02	-2.4e-02	5.6e-03	4.8e-04	-2.5e-03	-8.7e-04
SE	4.2e-03	4.2e-03	4.1e-03	5.6e-03	4.1e-03	4.4e-03	5.0e-03	4.1e-03	5.1e-03	5.8e-03	6.0e-03	7.8e-03	4.4e-03	1.8e-02	4.1e-03
**BMI**															
Beta	8.3e-03	4.0e-03	7.4e-02	9.4e-03	-4.8e-03	-5.7e-03	4.9e-03	-1.5e-03	2.3e-03	2.0e-04	-1.3e-03	8.1e-03	7.3e-04	5.2e-03	3.7e-05
SE	2.7e-03	2.7e-03	2.7e-03	3.6e-03	2.7e-03	2.9e-03	3.2e-03	2.7e-03	3.4e-03	3.9e-03	4.0e-03	5.1e-03	2.9e-03	1.2e-02	2.7e-03
**Total cholesterol**															
Beta	3.0e-02	-5.5e-03	-2.2e-02	5.4e-03	4.8e-03	-2.3e-02	-3.0e-03	-8.1e-03	5.2e-03	-7.2e-03	-6.7e-03	7.5e-03	1.3e-03	-8.4e-03	-4.1e-03
SE	3.1e-03	3.1e-03	3.1e-03	4.1e-03	3.0e-03	3.3e-03	3.7e-03	3.0e-03	3.8e-03	4.4e-03	4.5e-03	5.7e-03	3.2e-03	1.4e-02	3.0e-03
**Total cholesterol***															
Beta	2.5e-02	-3.6e-03	-1.1e-02	-2.9e-03	-4.4e-03	-1.5e-02	1.2e-03	-6.2e-03	7.8e-03	-1.1e-03	6.1e-04	4.1e-03	3.3e-04	-9.4e-03	-7.6e-03
SE	3.4e-03	3.4e-03	3.4e-03	4.5e-03	3.4e-03	3.7e-03	4.1e-03	3.4e-03	4.2e-03	4.9e-03	5.1e-03	6.4e-03	3.6e-03	1.5e-02	3.4e-03
**HDL-C**															
Beta	2.4e-02	-9.9e-04	-1.8e-02	7.7e-03	9.8e-04	-3.9e-03	8.0e-04	-6.0e-03	1.7e-04	-6.8e-03	-7.6e-03	-7.7e-03	-1.7e-03	2.0e-02	1.0e-03
SE	2.7e-03	2.6e-03	2.7e-03	3.5e-03	2.6e-03	2.9e-03	3.2e-03	2.6e-03	3.3e-03	3.8e-03	3.9e-03	5.0e-03	2.8e-03	1.2e-02	2.6e-03
**Triglycerides**															
Beta	-7.9e-03	1.7e-03	2.8e-03	-2.2e-03	9.4e-03	-7.5e-03	-3.8e-04	-1.6e-02	8.7e-04	5.9e-03	5.7e-03	6.9e-03	4.1e-03	-2.0e-02	-6.8e-03
SE	3.2e-03	3.2e-03	3.2e-03	4.2e-03	3.1e-03	3.4e-03	3.8e-03	3.2e-03	3.9e-03	4.5e-03	4.7e-03	5.9e-03	3.4e-03	1.4e-02	3.1e-03
**LDL-C**															
Beta	2.7e-02	-6.0e-03	-1.8e-02	3.7e-03	3.2e-03	-2.3e-02	-3.8e-03	-2.5e-03	5.7e-03	-8.2e-03	-7.1e-03	1.0e-02	1.1e-03	-1.2e-02	-3.0e-03
SE	3.1e-03	3.1e-03	3.1e-03	4.1e-03	3.0e-03	3.3e-03	3.7e-03	3.0e-03	3.8e-03	4.4e-03	4.5e-03	5.8e-03	3.3e-03	1.4e-02	3.0e-03
**LDL-C***															
Beta	2.2e-02	-3.5e-03	-7.1e-03	-5.3e-03	-6.1e-03	-1.4e-02	-3.8e-04	-1.4e-05	8.5e-03	-2.4e-03	-5.0e-05	6.8e-03	5.0e-04	-1.7e-02	-6.8e-03
SE	3.4e-03	3.4e-03	3.4e-03	4.6e-03	3.4e-03	3.7e-03	4.1e-03	3.4e-03	4.2e-03	4.9e-03	5.1e-03	6.4e-03	3.6e-03	1.5e-02	3.4e-03
**Lp(a)**															
Beta	2.3e-03	2.9e-03	-2.9e-03	4.8e-03	2.9e-03	4.5e-03	-6.6e-03	1.4e-03	3.6e-03	9.2e-03	1.1e-02	7.4e-03	-3.2e-03	-1.2e-02	3.0e-03
SE	3.5e-03	3.5e-03	3.5e-03	4.7e-03	3.5e-03	3.8e-03	4.2e-03	3.5e-03	4.4e-03	5.0e-03	5.2e-03	6.6e-03	3.7e-03	1.6e-02	3.5e-03
**Apolipoprotein A1**															
Beta	1.7e-02	2.8e-03	-1.2e-02	3.8e-03	2.7e-03	-5.2e-04	2.4e-03	-8.4e-03	-1.5e-03	-4.1e-03	-5.1e-03	-6.8e-03	-2.2e-03	2.4e-02	-8.6e-04
SE	2.8e-03	2.7e-03	2.8e-03	3.7e-03	2.7e-03	3.0e-03	3.3e-03	2.7e-03	3.4e-03	3.9e-03	4.1e-03	5.2e-03	2.9e-03	1.2e-02	2.7e-03

Tschiderer L, *et al. Heart* 2024; 110:710–717. doi: 10.1136/heartjnl-2023-323490

**Apolipoprotein B**															
Beta	2.1e-02	-5.0e-03	-1.4e-02	2.3e-03	2.6e-03	-2.2e-02	-2.3e-03	-2.4e-04	5.5e-03	-7.7e-03	-6.9e-03	7.8e-03	3.9e-04	-1.9e-02	-5.9e-03
SE	3.2e-03	3.1e-03	3.1e-03	4.2e-03	3.1e-03	3.4e-03	3.7e-03	3.1e-03	3.9e-03	4.5e-03	4.6e-03	5.8e-03	3.3e-03	1.4e-02	3.1e-03
**HbA1c**															
Beta	1.6e-02	1.3e-03	3.2e-02	-3.0e-03	2.1e-04	-6.3e-03	6.3e-03	1.5e-02	-2.5e-03	3.5e-03	3.7e-03	9.9e-03	6.9e-04	1.9e-03	-7.5e-03
SE	3.5e-03	3.4e-03	3.5e-03	4.6e-03	3.4e-03	3.7e-03	4.1e-03	3.4e-03	4.3e-03	4.9e-03	5.1e-03	6.4e-03	3.7e-03	1.6e-02	3.4e-03
**Creatinine**															
Beta	-6.8e-03	5.2e-04	-7.2e-03	-1.7e-03	8.0e-04	-1.6e-02	7.9e-05	4.0e-03	1.8e-03	-2.7e-03	-3.5e-03	-3.7e-04	9.5e-04	-1.0e-02	6.6e-03
SE	2.6e-03	2.5e-03	2.6e-03	3.4e-03	2.5e-03	2.8e-03	3.0e-03	2.5e-03	3.2e-03	3.6e-03	3.8e-03	4.8e-03	2.7e-03	1.1e-02	2.5e-03
**Albumin**															
Beta	2.5e-02	7.2e-03	-9.9e-03	-6.6e-03	1.0e-03	4.5e-03	-7.7e-03	2.6e-03	3.3e-03	1.2e-03	1.6e-03	-7.6e-03	-6.2e-03	2.6e-02	-3.6e-05
SE	3.2e-03	3.1e-03	3.2e-03	4.2e-03	3.1e-03	3.4e-03	3.8e-03	3.1e-03	3.9e-03	4.5e-03	4.6e-03	5.9e-03	3.3e-03	1.4e-02	3.1e-03
**ALAT**															
Beta	-2.5e-02	2.3e-03	2.2e-02	-1.3e-02	9.6e-04	-1.4e-03	6.6e-03	2.6e-03	4.0e-04	-1.3e-03	-2.7e-03	1.5e-04	9.0e-04	5.7e-03	-2.1e-03
SE	3.0e-03	3.0e-03	3.0e-03	4.0e-03	2.9e-03	3.2e-03	3.6e-03	3.0e-03	3.7e-03	4.3e-03	4.4e-03	5.6e-03	3.2e-03	1.3e-02	2.9e-03
**ASAT**															
Beta	-1.7e-02	1.2e-03	1.6e-02	-2.9e-05	-4.8e-04	3.7e-03	2.6e-03	-1.0e-02	-3.6e-03	-1.3e-03	-2.1e-03	-7.3e-03	2.5e-03	2.2e-03	-2.5e-03
SE	3.2e-03	3.1e-03	3.2e-03	4.2e-03	3.1e-03	3.4e-03	3.7e-03	3.1e-03	3.9e-03	4.5e-03	4.6e-03	5.9e-03	3.3e-03	1.4e-02	3.1e-03
**GGT**															
Beta	-1.5e-03	4.8e-03	4.3e-03	-5.1e-03	1.1e-04	-4.1e-03	1.2e-02	-9.6e-03	1.5e-03	-5.6e-04	-2.8e-04	-1.0e-02	2.1e-03	8.9e-03	-6.3e-03
SE	3.0e-03	3.0e-03	3.0e-03	4.0e-03	3.0e-03	3.3e-03	3.6e-03	3.0e-03	3.7e-03	4.3e-03	4.4e-03	5.6e-03	3.2e-03	1.3e-02	3.0e-03
**CRP**															
Beta	-1.3e-02	1.5e-03	2.1e-02	-9.7e-04	-1.8e-03	-7.2e-03	1.0e-02	-5.4e-03	-7.3e-03	-2.5e-05	4.7e-03	1.5e-03	1.4e-03	-1.7e-02	-4.8e-03
SE	3.0e-03	3.0e-03	3.0e-03	4.0e-03	3.0e-03	3.2e-03	3.6e-03	3.0e-03	3.7e-03	4.3e-03	4.4e-03	5.6e-03	3.2e-03	1.3e-02	3.0e-03

*Restricted to individuals not taking lipid-lowering therapy. Results are from linear regression for cardiovascular risk factors and from logistic regression for cardiovascular events. Models were adjusted for age at baseline and the first 16 genetic principal components. The variables triglycerides, Lp(a), creatinine, ALAT, ASAT, GGT, and CRP were log-transformed. Abbreviations: ALAT, alanine aminotransferase; ASAT, aspartate aminotransferase; BMI, body mass index; CRP, C-reactive protein; CVD, cardiovascular disease; DBP, diastolic blood pressure; GGT, gamma glutamyltransferase; GH, gestational hypertension; HbA1c, glycated haemoglobin; HDL-C, high-density lipoprotein cholesterol; ICH, intracerebral haemorrhage LDL-C, low-density lipoprotein cholesterol; Lp(a), lipoprotein(a); MI, myocardial infarction; PE, pre-eclampsia/eclampsia; SAH, subarachnoid haemorrhage; SBP, systolic blood pressure SE, standard error; SNP, single nucleotide polymorphism.

Table S4. Heterogeneity statistics for the Mendelian Randomisation analysis using inverse-variance weighted regression in ever pregnant women.

Outcome	Cochran's Q statistic	P-value
**Pre-eclampsia/eclampsia**		
CVD	28.5	0.002
MI	34.3	<0.001
Stroke	17.7	0.060
Ischaemic stroke	15.0	0.132
Haemorrhagic stroke	8.9	0.544
ICH	8.7	0.562
SAH	10.1	0.434
Ischaemic CVD	30.3	<0.001
**Gestational hypertension**		
CVD	4.1	0.541
MI	3.9	0.557
Stroke	8.5	0.131
Ischaemic stroke	8.5	0.131
Haemorrhagic stroke	0.6	0.986
ICH	1.1	0.950
SAH	1.5	0.917
Ischaemic CVD	2.8	0.738

Abbreviations: CVD, cardiovascular disease; ICH, intracerebral haemorrhage; MI, myocardial infarction; SAH, subarachnoid haemorrhage.

Tschiderer L, *et al. Heart* 2024; 110:710–717. doi: 10.1136/heartjnl-2023-323490

Table S5. MR-PRESSO in ever pregnant women.

Outcome	Primary analysis (IVW)		MR-PRESSO			
	OR (95% CI)	P-value	SNPs excluded	OR (95% CI)	P-value	P-value distortion test*
**Pre-eclampsia/eclampsia**						
CVD	1.20 (1.02, 1.41)	0.026	rs10774624, rs7318880	1.20 (1.07, 1.36)	0.002	0.935
MI	1.20 (0.94, 1.52)	0.144	rs10774624, rs7318880	1.18 (1.00, 1.38)	0.044	0.790
Stroke	1.20 (1.01, 1.42)	0.038	–	–	–	–
Ischaemic stroke	1.29 (1.04, 1.60)	0.018	–	–	–	–
Haemorrhagic stroke	1.09 (0.85, 1.40)	0.483	–	–	–	–
ICH	0.94 (0.67, 1.33)	0.736	–	–	–	–
SAH	1.13 (0.82, 1.57)	0.462	–	–	–	–
Ischaemic CVD	1.23 (1.02, 1.48)	0.034	rs10774624, rs7318880	1.20 (1.06, 1.36)	0.003	0.698
**Gestational hypertension**						
CVD	1.24 (1.12, 1.38)	<0.001	–	–	–	–
MI	1.25 (1.09, 1.44)	0.002	–	–	–	–
Stroke	1.26 (1.05, 1.52)	0.013	–	–	–	–
Ischaemic stroke	1.34 (1.05, 1.72)	0.021	–	–	–	–
Haemorrhagic stroke	1.43 (1.09, 1.87)	0.009	–	–	–	–
ICH	1.36 (0.94, 1.98)	0.105	–	–	–	–
SAH	1.51 (1.06, 2.15)	0.022	–	–	–	–
Ischaemic CVD	1.25 (1.11, 1.41)	<0.001	–	–	–	–

*The distortion test tests the difference in the estimates before and after correction for outliers. Abbreviations: CI, confidence interval; CVD, cardiovascular disease; OR, odds ratio; ICH, intracerebral haemorrhage; IVW, inverse-variance weighting; MI, myocardial infarction; SAH, subarachnoid haemorrhage; SNP, single nucleotide polymorphism.

Tschiderer L, *et al. Heart* 2024; 110:710–717. doi: 10.1136/heartjnl-2023-323490

Supplementary Figures

Figure S1. Flow chart for the selection of SNPs included in the primary analysis.

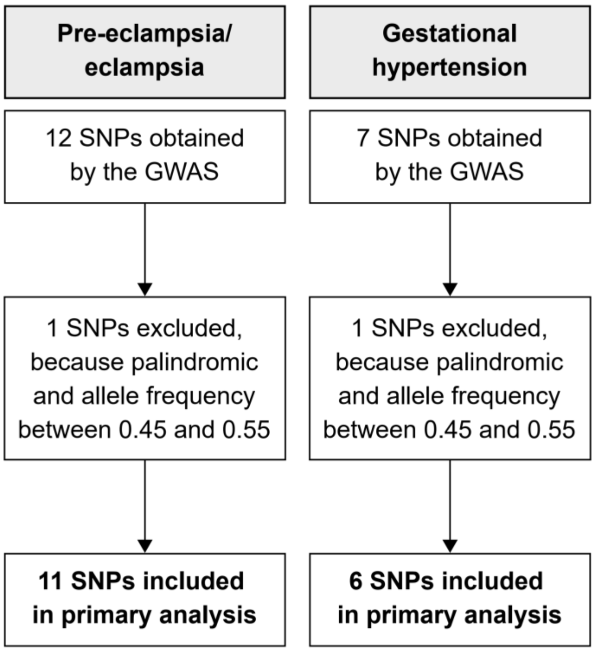

Abbreviations: GWAS, genome-wide association study; SNP, single nucleotide polymorphism.

Tschiderer L, *et al. Heart* 2024; 110:710–717. doi: 10.1136/heartjnl-2023-323490

Figure S2. Mendelian Randomisation analysis of genetic liability to <u>pre-eclampsia/eclampsia</u> and risk of cardiovascular events in <u>ever pregnant women</u> based on <u>different methods</u>.

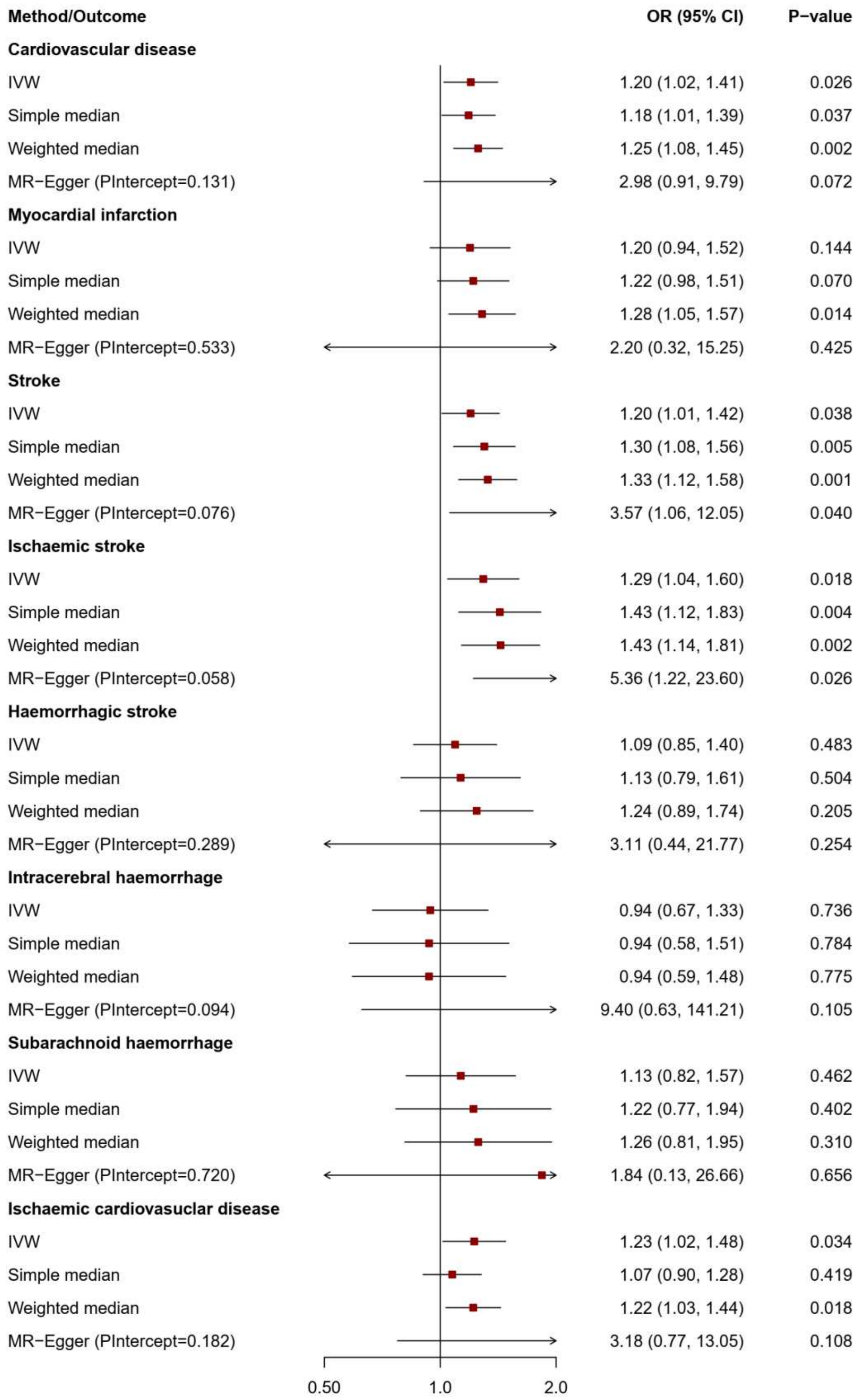

Models were adjusted for age at baseline and the first 16 genetic principal components. Abbreviations: IVW, inverse-variance weighted; CI, confidence interval; OR, odds ratio.

Tschiderer L, *et al. Heart* 2024; 110:710–717. doi: 10.1136/heartjnl-2023-323490

Figure S3. Mendelian Randomisation analysis of genetic liability to gestational hypertension and risk of cardiovascular events in ever pregnant women based on different methods.

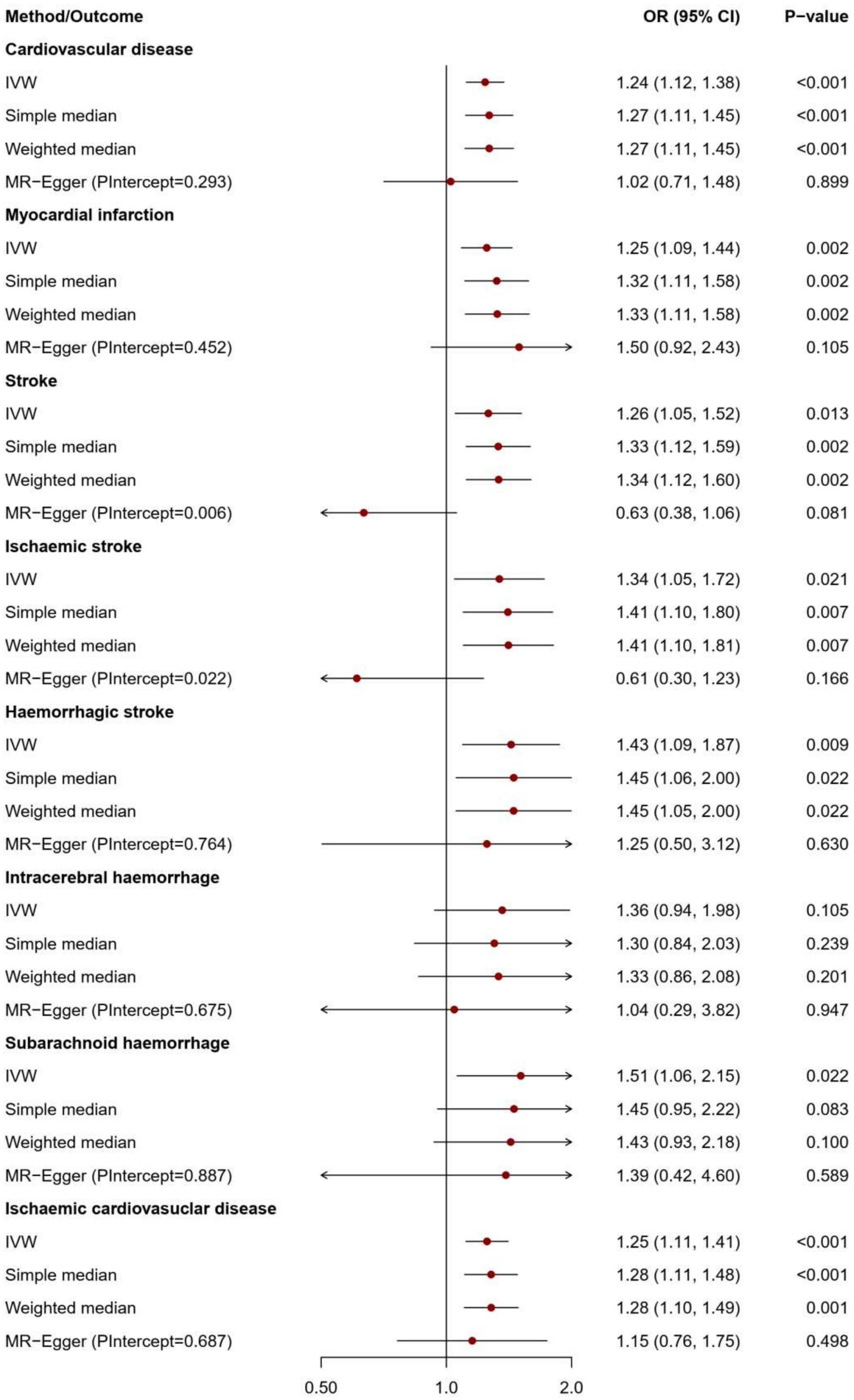

Models were adjusted for age at baseline and the first 16 genetic principal components. Abbreviations: IVW, inverse-variance weighted; CI, confidence interval; OR, odds ratio.

Tschiderer L, *et al. Heart* 2024; 110:710–717. doi: 10.1136/heartjnl-2023-323490

Figure S4. Mendelian Randomisation analysis of genetic liability to pre-eclampsia/eclampsia and risk of cardiovascular events in ever pregnant women based on Cox-regression analysis.

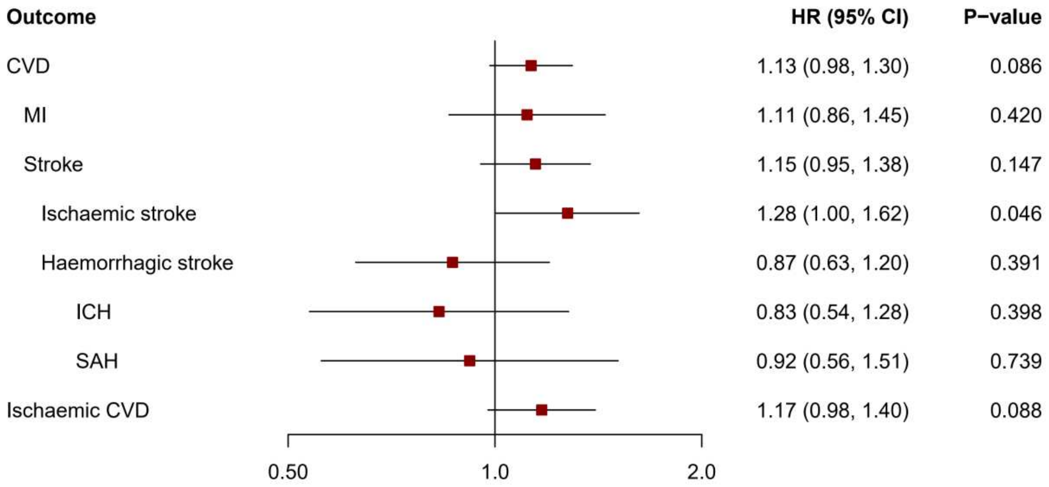

Results are from inverse-variance weighted regression. Models were adjusted for the first 16 genetic principal components. Abbreviations: CI, confidence interval; CVD, cardiovascular disease; HR, hazard ratio; ICH, intracerebral haemorrhage; MI, myocardial infarction; SAH, subarachnoid haemorrhage.

Tschiderer L, *et al. Heart* 2024; 110:710–717. doi: 10.1136/heartjnl-2023-323490

Figure S5. Mendelian Randomisation analysis of genetic liability to gestational hypertension and risk of cardiovascular events in ever pregnant women based on Cox-regression analysis.

Outcome	HR (95% CI)	P−value
CVD	1.21 (1.07, 1.38)	0.003
MI	1.21 (1.02, 1.44)	0.030
Stroke	1.22 (1.01, 1.47)	0.042
Ischaemic stroke	1.24 (0.99, 1.54)	0.061
Haemorrhagic stroke	1.21 (0.85, 1.72)	0.291
ICH	1.23 (0.77, 1.96)	0.397
SAH	1.22 (0.71, 2.08)	0.466
Ischaemic CVD	1.20 (1.05, 1.38)	0.008

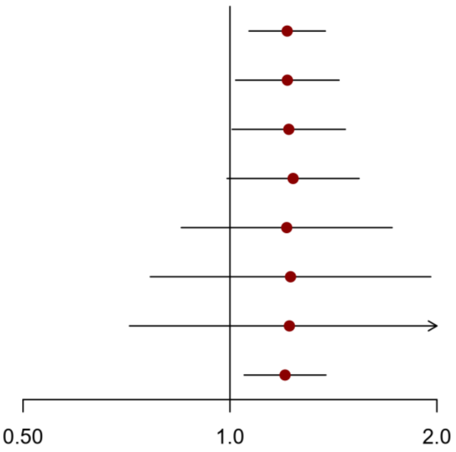

Results are from inverse-variance weighted regression. Models were adjusted for the first 16 genetic principal components. Abbreviations: CI, confidence interval; CVD, cardiovascular disease; HR, hazard ratio; ICH, intracerebral haemorrhage; MI, myocardial infarction; SAH, subarachnoid haemorrhage.

Tschiderer L, *et al. Heart* 2024; 110:710–717. doi: 10.1136/heartjnl-2023-323490

Figure S6. Mendelian Randomisation analysis of genetic liability to pre-eclampsia/eclampsia and risk of cardiovascular events in ever pregnant women additionally adjusted for phenotypic systolic blood pressure levels.

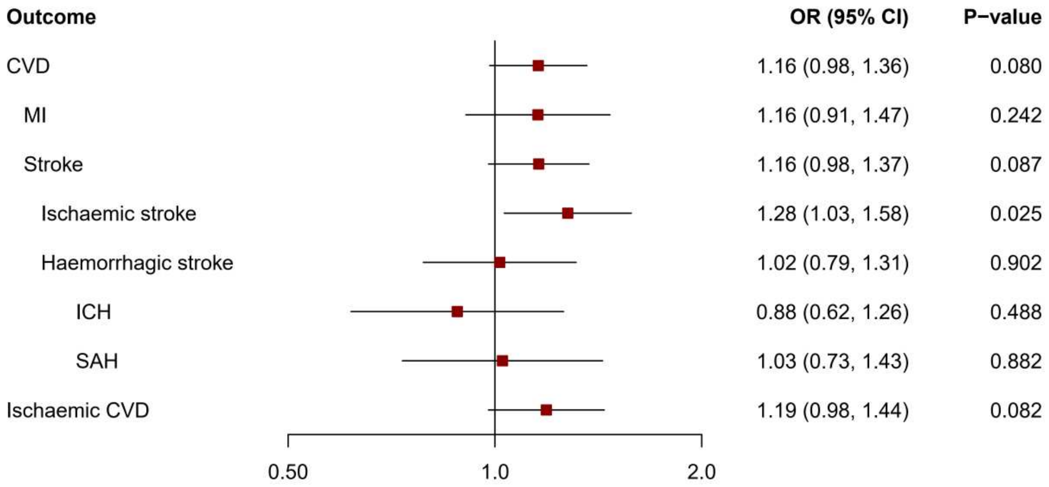

Results are from inverse-variance weighted regression. Models were adjusted for age at baseline, systolic blood pressure at baseline, and the first 16 genetic principal components. Abbreviations: CI, confidence interval; CVD, cardiovascular disease; ICH, intracerebral haemorrhage; MI, myocardial infarction; OR, odds ratio; SAH, subarachnoid haemorrhage.

Tschiderer L, *et al. Heart* 2024; 110:710–717. doi: 10.1136/heartjnl-2023-323490

Figure S7. Mendelian Randomisation analysis of genetic liability to gestational hypertension and risk of cardiovascular events in ever pregnant women additionally adjusted for phenotypic systolic blood pressure levels.

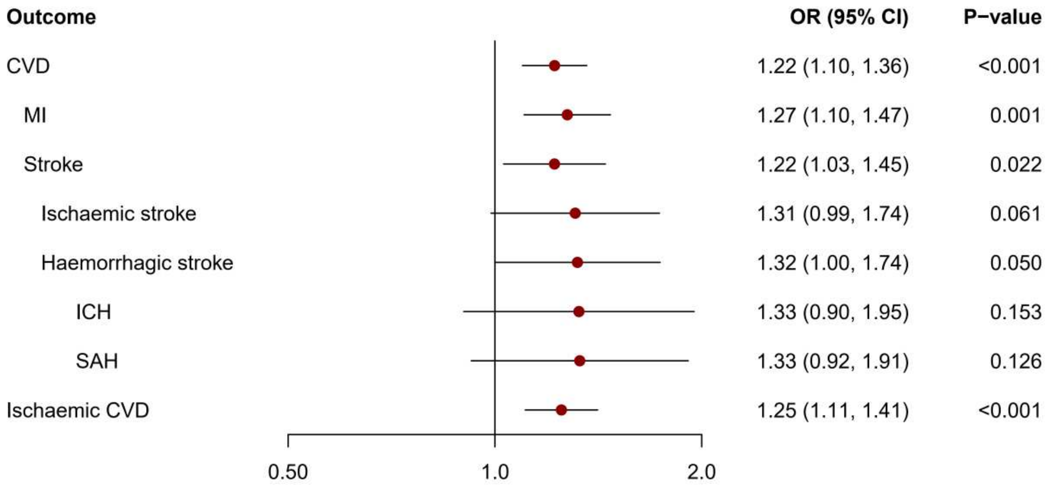

Results are from inverse-variance weighted regression. Models were adjusted for age at baseline, systolic blood pressure at baseline, and the first 16 genetic principal components. Abbreviations: CI, confidence interval; CVD, cardiovascular disease; ICH, intracerebral haemorrhage; MI, myocardial infarction; OR, odds ratio; SAH, subarachnoid haemorrhage.

Tschiderer L, *et al. Heart* 2024; 110:710–717. doi: 10.1136/heartjnl-2023-323490

Figure S8. Mendelian Randomisation analysis of genetic liability to pre-eclampsia/eclampsia and risk of cardiovascular events in never pregnant women.

Outcome	OR (95% CI)	P−value	Ratio of ORs* (95% CI)	P−value*
CVD	1.22 (0.90, 1.65)	0.195	1.02 (0.72, 1.43)	0.925
MI	1.44 (0.89, 2.33)	0.141	1.20 (0.70, 2.06)	0.501
Stroke	1.01 (0.72, 1.40)	0.971	0.84 (0.58, 1.21)	0.352
Ischaemic stroke	1.30 (0.81, 2.08)	0.272	1.01 (0.60, 1.69)	0.980
Haemorrhagic stroke	0.83 (0.44, 1.55)	0.562	0.76 (0.39, 1.49)	0.425
ICH	1.42 (0.54, 3.73)	0.477	1.51 (0.54, 4.21)	0.433
SAH	0.57 (0.25, 1.29)	0.176	0.50 (0.21, 1.21)	0.126
Ischaemic CVD	1.40 (0.95, 2.06)	0.091	1.14 (0.74, 1.76)	0.548

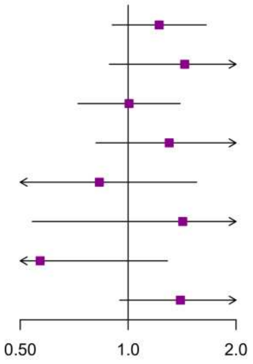

*As compared to ever pregnant women. Results are from inverse-variance weighted regression. Models were adjusted for age at baseline and the first 16 genetic principal components. Abbreviations: CI, confidence interval; CVD, cardiovascular disease; ICH, intracerebral haemorrhage; MI, myocardial infarction; OR, odds ratio; SAH, subarachnoid haemorrhage.

Tschiderer L, *et al. Heart* 2024; 110:710–717. doi: 10.1136/heartjnl-2023-323490

Figure S9. Mendelian Randomisation analysis of genetic liability to gestational hypertension and risk of cardiovascular events in never pregnant women.

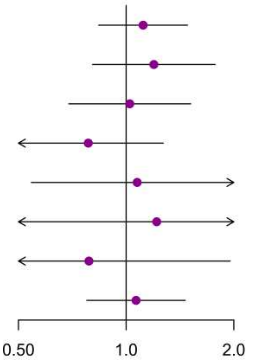

Outcome	OR (95% CI)	P−value	Ratio of ORs* (95% CI)	P−value*
CVD	1.12 (0.84, 1.48)	0.454	0.90 (0.66, 1.22)	0.498
MI	1.20 (0.81, 1.77)	0.375	0.96 (0.63, 1.45)	0.833
Stroke	1.02 (0.69, 1.52)	0.907	0.81 (0.53, 1.25)	0.344
Ischaemic stroke	0.78 (0.48, 1.27)	0.322	0.58 (0.34, 1.01)	0.053
Haemorrhagic stroke	1.07 (0.54, 2.12)	0.838	0.75 (0.36, 1.56)	0.442
ICH	1.22 (0.46, 3.19)	0.689	0.89 (0.32, 2.51)	0.831
SAH	0.79 (0.32, 1.95)	0.606	0.52 (0.20, 1.38)	0.190
Ischaemic CVD	1.07 (0.78, 1.46)	0.694	0.85 (0.61, 1.19)	0.350

*As compared to ever pregnant women. Results are from inverse-variance weighted regression. Models were adjusted for age at baseline and the first 16 genetic principal components. Abbreviations: CI, confidence interval; CVD, cardiovascular disease; ICH, intracerebral haemorrhage; MI, myocardial infarction; OR, odds ratio; SAH, subarachnoid haemorrhage.

Tschiderer L, *et al. Heart* 2024; 110:710–717. doi: 10.1136/heartjnl-2023-323490

Figure S10. Mendelian Randomisation analysis of genetic liability to <u>pre-eclampsia/eclampsia</u> and risk of cardiovascular events in <u>men</u>.

Outcome	OR (95% CI)	P−value	Ratio of ORs* (95% CI)	P−value*
CVD	1.29 (1.08, 1.53)	0.004	1.07 (0.84, 1.36)	0.572
MI	1.33 (1.12, 1.57)	0.001	1.11 (0.83, 1.49)	0.488
Stroke	1.25 (1.07, 1.45)	0.004	1.04 (0.83, 1.31)	0.734
Ischaemic stroke	1.33 (1.09, 1.63)	0.006	1.03 (0.77, 1.38)	0.853
Haemorrhagic stroke	0.90 (0.70, 1.16)	0.428	0.82 (0.58, 1.18)	0.290
ICH	0.91 (0.66, 1.25)	0.548	0.96 (0.60, 1.54)	0.874
SAH	0.83 (0.57, 1.22)	0.354	0.74 (0.45, 1.22)	0.237
Ischaemic CVD	1.32 (1.10, 1.59)	0.003	1.08 (0.83, 1.41)	0.565

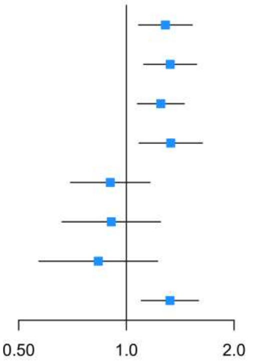

*As compared to ever pregnant women. Results are from inverse-variance weighted regression. Models were adjusted for age at baseline and the first 16 genetic principal components. Abbreviations: CI, confidence interval; CVD, cardiovascular disease; ICH, intracerebral haemorrhage; MI, myocardial infarction; OR, odds ratio; SAH, subarachnoid haemorrhage.

Tschiderer L, *et al. Heart* 2024; 110:710–717. doi: 10.1136/heartjnl-2023-323490

Figure S11. Mendelian Randomisation analysis of genetic liability to gestational hypertension and risk of cardiovascular events in men.

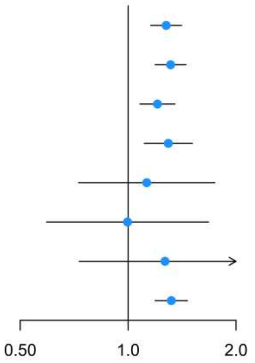

Outcome	OR (95% CI)	P−value	Ratio of ORs* (95% CI)	P−value*
CVD	1.28 (1.16, 1.41)	<0.001	1.03 (0.89, 1.19)	0.686
MI	1.31 (1.19, 1.45)	<0.001	1.05 (0.88, 1.25)	0.573
Stroke	1.21 (1.08, 1.35)	0.001	0.96 (0.77, 1.19)	0.689
Ischaemic stroke	1.29 (1.11, 1.51)	0.001	0.97 (0.72, 1.29)	0.813
Haemorrhagic stroke	1.13 (0.73, 1.75)	0.593	0.79 (0.47, 1.32)	0.361
ICH	1.00 (0.59, 1.68)	0.989	0.73 (0.39, 1.39)	0.339
SAH	1.27 (0.73, 2.20)	0.399	0.84 (0.44, 1.62)	0.600
Ischaemic CVD	1.32 (1.19, 1.46)	<0.001	1.05 (0.90, 1.23)	0.511

*As compared to ever pregnant women. Results are from inverse-variance weighted regression. Models were adjusted for age at baseline and the first 16 genetic principal components. Abbreviations: CI, confidence interval; CVD, cardiovascular disease; ICH, intracerebral haemorrhage; MI, myocardial infarction; OR, odds ratio; SAH, subarachnoid haemorrhage.

Tschiderer L, *et al. Heart* 2024; 110:710–717. doi: 10.1136/heartjnl-2023-323490

Figure S12. Mendelian Randomisation analysis of genetic liability to pre-eclampsia/eclampsia and cardiovascular risk factors in never pregnant women.

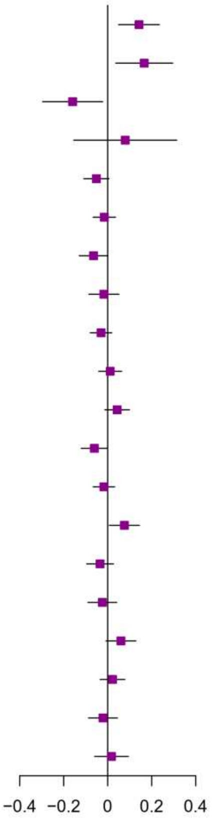

Outcome	Beta (95% CI)	P−value	Difference* (95% CI)	P−value*
Systolic blood pressure	0.14 (0.05, 0.24)	0.003	−0.02 (−0.16, 0.12)	0.781
Diastolic blood pressure	0.17 (0.04, 0.30)	0.012	0.01 (−0.16, 0.18)	0.897
Age at hypertension	−0.16 (−0.30, −0.02)	0.022	0.07 (−0.10, 0.24)	0.435
Body mass index	0.08 (−0.15, 0.31)	0.504	0.02 (−0.26, 0.30)	0.894
Total cholesterol	−0.05 (−0.11, 0.01)	0.080	0.01 (−0.07, 0.10)	0.752
Total cholesterol†	−0.02 (−0.07, 0.04)	0.555	0.03 (−0.06, 0.11)	0.529
HDL cholesterol	−0.06 (−0.13, 0.00)	0.053	−0.02 (−0.11, 0.06)	0.567
Triglycerides	−0.02 (−0.09, 0.05)	0.622	0.00 (−0.07, 0.08)	0.956
LDL cholesterol	−0.03 (−0.08, 0.02)	0.227	0.03 (−0.05, 0.10)	0.517
LDL cholesterol†	0.01 (−0.04, 0.06)	0.669	0.04 (−0.04, 0.12)	0.330
Lipoprotein(a)	0.04 (−0.01, 0.10)	0.133	0.04 (−0.02, 0.11)	0.175
Apolipoprotein A1	−0.06 (−0.12, −0.00)	0.048	−0.04 (−0.12, 0.03)	0.262
Apolipoprotein B	−0.02 (−0.07, 0.03)	0.497	0.02 (−0.04, 0.09)	0.501
HbA1c	0.08 (0.01, 0.14)	0.030	0.08 (−0.02, 0.17)	0.105
Creatinine	−0.03 (−0.10, 0.03)	0.265	−0.01 (−0.08, 0.07)	0.864
Albumin	−0.02 (−0.09, 0.04)	0.467	0.00 (−0.08, 0.09)	0.931
ALAT	0.06 (−0.01, 0.13)	0.083	0.01 (−0.08, 0.10)	0.812
ASAT	0.02 (−0.03, 0.08)	0.443	0.01 (−0.07, 0.09)	0.871
GGT	−0.02 (−0.09, 0.04)	0.526	−0.02 (−0.09, 0.05)	0.548
C−reactive protein	0.02 (−0.06, 0.09)	0.660	−0.01 (−0.11, 0.08)	0.775

*As compared to ever pregnant women. †Restricted to individuals not taking lipid-lowering therapy. Results are from inverse-variance weighted regression. Models were adjusted for age at baseline and the first 16 genetic principal components. The variables triglycerides, lipoprotein(a), creatinine, ALAT, ASAT, GGT, and C-reactive protein were log-transformed. Abbreviations: ALAT, alanine aminotransferase; ASAT, aspartate aminotransferase; CI, confidence interval; GGT, gamma glutamyltransferase; HbA1c, glycated haemoglobin; HDL, high-density lipoprotein; LDL, low-density lipoprotein.

Tschiderer L, *et al. Heart* 2024; 110:710–717. doi: 10.1136/heartjnl-2023-323490

Figure S13. Mendelian Randomisation analysis of genetic liability to <u>gestational hypertension</u> and cardiovascular risk factors in <u>never pregnant women</u>.

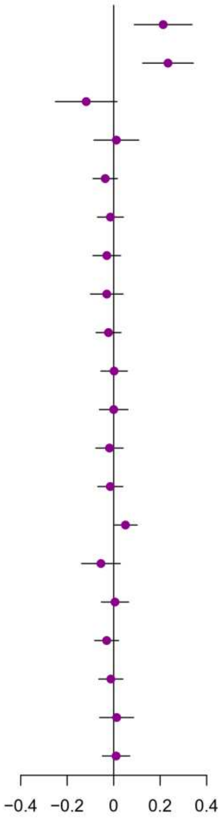

Outcome	Beta (95% CI)	P−value	Difference* (95% CI)	P−value*
Systolic blood pressure	0.21 (0.09, 0.34)	<0.001	−0.01 (−0.16, 0.15)	0.949
Diastolic blood pressure	0.23 (0.12, 0.34)	<0.001	0.05 (−0.09, 0.19)	0.484
Age at hypertension	−0.12 (−0.25, 0.01)	0.081	0.11 (−0.07, 0.29)	0.247
Body mass index	0.01 (−0.08, 0.11)	0.814	0.01 (−0.09, 0.11)	0.860
Total cholesterol	−0.04 (−0.09, 0.02)	0.176	−0.02 (−0.09, 0.05)	0.588
Total cholesterol†	−0.01 (−0.07, 0.04)	0.624	−0.02 (−0.09, 0.05)	0.518
HDL cholesterol	−0.03 (−0.09, 0.03)	0.331	−0.01 (−0.08, 0.06)	0.778
Triglycerides	−0.03 (−0.10, 0.04)	0.409	−0.03 (−0.10, 0.05)	0.503
LDL cholesterol	−0.02 (−0.08, 0.03)	0.416	−0.01 (−0.09, 0.06)	0.753
LDL cholesterol†	0.00 (−0.06, 0.06)	0.946	−0.02 (−0.10, 0.06)	0.634
Lipoprotein(a)	0.00 (−0.06, 0.06)	0.995	−0.01 (−0.07, 0.06)	0.861
Apolipoprotein A1	−0.02 (−0.08, 0.04)	0.551	−0.00 (−0.08, 0.07)	0.916
Apolipoprotein B	−0.01 (−0.07, 0.04)	0.598	−0.01 (−0.08, 0.06)	0.684
HbA1c	0.05 (0.00, 0.10)	0.048	0.06 (0.00, 0.11)	0.041
Creatinine	−0.05 (−0.14, 0.03)	0.196	−0.01 (−0.11, 0.09)	0.849
Albumin	0.01 (−0.05, 0.06)	0.849	−0.00 (−0.07, 0.06)	0.906
ALAT	−0.03 (−0.08, 0.02)	0.252	−0.05 (−0.12, 0.03)	0.208
ASAT	−0.01 (−0.06, 0.04)	0.646	−0.02 (−0.09, 0.05)	0.614
GGT	0.01 (−0.06, 0.09)	0.731	−0.00 (−0.09, 0.08)	0.944
C−reactive protein	0.01 (−0.05, 0.07)	0.728	0.00 (−0.06, 0.06)	0.990

*As compared to ever pregnant women. †Restricted to individuals not taking lipid-lowering therapy. Results are from inverse-variance weighted regression. Models were adjusted for age at baseline and the first 16 genetic principal components. The variables triglycerides, lipoprotein(a), creatinine, ALAT, ASAT, GGT, and C-reactive protein were log-transformed. Abbreviations: ALAT, alanine aminotransferase; ASAT, aspartate aminotransferase; CI, confidence interval; GGT, gamma glutamyltransferase; HbA1c, glycated haemoglobin; HDL, high-density lipoprotein; LDL, low-density lipoprotein.

Tschiderer L, *et al. Heart* 2024; 110:710–717. doi: 10.1136/heartjnl-2023-323490

Figure S14. Mendelian Randomisation analysis of genetic liability to pre-eclampsia/eclampsia and cardiovascular risk factors in men.

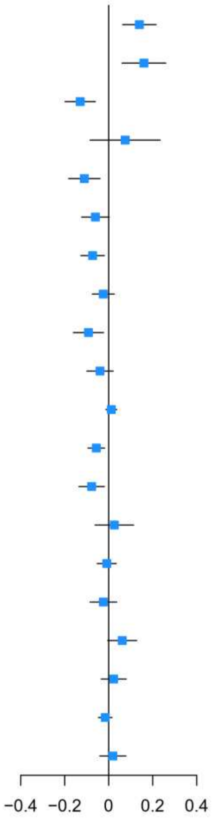

Outcome	Beta (95% CI)	P−value	Difference* (95% CI)	P−value*
Systolic blood pressure	0.14 (0.06, 0.22)	<0.001	−0.02 (−0.15, 0.11)	0.732
Diastolic blood pressure	0.16 (0.06, 0.26)	0.002	0.01 (−0.14, 0.15)	0.943
Age at hypertension	−0.13 (−0.20, −0.06)	<0.001	0.10 (−0.02, 0.22)	0.117
Body mass index	0.07 (−0.08, 0.23)	0.356	0.01 (−0.21, 0.24)	0.900
Total cholesterol	−0.11 (−0.18, −0.04)	0.002	−0.05 (−0.14, 0.05)	0.344
Total cholesterol†	−0.06 (−0.12, 0.00)	0.059	−0.02 (−0.11, 0.07)	0.698
HDL cholesterol	−0.07 (−0.13, −0.02)	0.007	−0.03 (−0.11, 0.04)	0.384
Triglycerides	−0.02 (−0.07, 0.03)	0.333	−0.01 (−0.07, 0.06)	0.867
LDL cholesterol	−0.09 (−0.16, −0.02)	0.009	−0.04 (−0.13, 0.05)	0.432
LDL cholesterol†	−0.04 (−0.10, 0.02)	0.197	−0.01 (−0.10, 0.08)	0.817
Lipoprotein(a)	0.01 (−0.01, 0.04)	0.347	0.01 (−0.02, 0.05)	0.520
Apolipoprotein A1	−0.06 (−0.09, −0.02)	0.004	−0.04 (−0.10, 0.02)	0.196
Apolipoprotein B	−0.08 (−0.14, −0.02)	0.009	−0.04 (−0.11, 0.04)	0.334
HbA1c	0.03 (−0.06, 0.11)	0.569	0.02 (−0.08, 0.13)	0.647
Creatinine	−0.01 (−0.05, 0.03)	0.693	0.02 (−0.04, 0.08)	0.556
Albumin	−0.02 (−0.08, 0.04)	0.434	0.00 (−0.08, 0.09)	0.926
ALAT	0.06 (−0.00, 0.13)	0.068	0.01 (−0.07, 0.10)	0.787
ASAT	0.02 (−0.03, 0.08)	0.438	0.01 (−0.07, 0.09)	0.858
GGT	−0.02 (−0.05, 0.02)	0.322	−0.02 (−0.06, 0.03)	0.450
C−reactive protein	0.02 (−0.04, 0.08)	0.534	−0.01 (−0.09, 0.07)	0.770

*As compared to ever pregnant women. †Restricted to individuals not taking lipid-lowering therapy. Results are from inverse-variance weighted regression. Models were adjusted for age at baseline and the first 16 genetic principal components. The variables triglycerides, lipoprotein(a), creatinine, ALAT, ASAT, GGT, and C-reactive protein were log-transformed. Abbreviations: ALAT, alanine aminotransferase; ASAT, aspartate aminotransferase; CI, confidence interval; GGT, gamma glutamyltransferase; HbA1c, glycated haemoglobin; HDL, high-density lipoprotein; LDL, low-density lipoprotein.

Tschiderer L, *et al. Heart* 2024; 110:710–717. doi: 10.1136/heartjnl-2023-323490

Figure S15. Mendelian Randomisation analysis of genetic liability to gestational hypertension and cardiovascular risk factors in men.

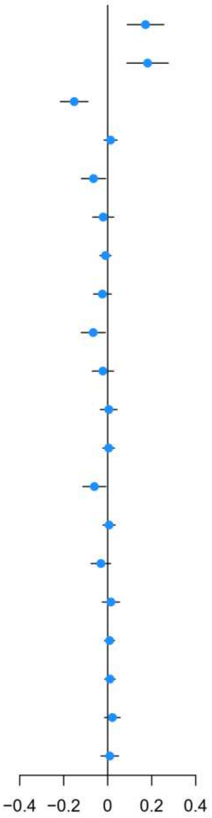

Outcome	Beta (95% CI)	P−value	Difference* (95% CI)	P−value*
Systolic blood pressure	0.17 (0.09, 0.26)	<0.001	−0.05 (−0.18, 0.08)	0.482
Diastolic blood pressure	0.18 (0.09, 0.28)	<0.001	−0.00 (−0.13, 0.13)	0.980
Age at hypertension	−0.15 (−0.21, −0.09)	<0.001	0.07 (−0.07, 0.21)	0.302
Body mass index	0.01 (−0.02, 0.04)	0.384	0.01 (−0.03, 0.05)	0.593
Total cholesterol	−0.06 (−0.12, −0.01)	0.022	−0.05 (−0.12, 0.02)	0.197
Total cholesterol†	−0.02 (−0.07, 0.03)	0.402	−0.03 (−0.10, 0.04)	0.368
HDL cholesterol	−0.01 (−0.04, 0.02)	0.446	0.01 (−0.03, 0.05)	0.660
Triglycerides	−0.02 (−0.06, 0.02)	0.242	−0.02 (−0.07, 0.03)	0.423
LDL cholesterol	−0.07 (−0.12, −0.01)	0.019	−0.06 (−0.13, 0.02)	0.153
LDL cholesterol†	−0.02 (−0.07, 0.03)	0.378	−0.04 (−0.11, 0.03)	0.242
Lipoprotein(a)	0.00 (−0.03, 0.04)	0.797	−0.00 (−0.05, 0.05)	0.960
Apolipoprotein A1	0.00 (−0.02, 0.03)	0.740	0.02 (−0.04, 0.07)	0.510
Apolipoprotein B	−0.06 (−0.11, −0.01)	0.023	−0.06 (−0.13, 0.01)	0.084
HbA1c	0.01 (−0.02, 0.03)	0.654	0.01 (−0.02, 0.05)	0.476
Creatinine	−0.03 (−0.08, 0.01)	0.175	0.01 (−0.06, 0.09)	0.701
Albumin	0.01 (−0.02, 0.05)	0.470	0.00 (−0.05, 0.06)	0.863
ALAT	0.01 (−0.01, 0.03)	0.461	−0.01 (−0.06, 0.05)	0.812
ASAT	0.01 (−0.01, 0.03)	0.397	0.00 (−0.04, 0.05)	0.844
GGT	0.02 (−0.01, 0.06)	0.238	0.01 (−0.05, 0.06)	0.839
C−reactive protein	0.01 (−0.03, 0.05)	0.644	−0.00 (−0.05, 0.05)	0.980

*As compared to ever pregnant women. †Restricted to individuals not taking lipid-lowering therapy. Results are from inverse-variance weighted regression. Models were adjusted for age at baseline and the first 16 genetic principal components. The variables triglycerides, lipoprotein(a), creatinine, ALAT, ASAT, GGT, and C-reactive protein were log-transformed. Abbreviations: ALAT, alanine aminotransferase; ASAT, aspartate aminotransferase; CI, confidence interval; GGT, gamma glutamyltransferase; HbA1c, glycated haemoglobin; HDL, high-density lipoprotein; LDL, low-density lipoprotein.

Tschiderer L, *et al. Heart* 2024; 110:710–717. doi: 10.1136/heartjnl-2023-323490
